# Control of membrane protein homeostasis by a chaperone-like glial cell adhesion molecule at multiple subcellular locations

**DOI:** 10.1038/s41598-021-97777-4

**Published:** 2021-09-16

**Authors:** Haijin Xu, Sandra Isenmann, Tania López-Hernández, Raúl Estévez, Gergely L. Lukacs, Pirjo M. Apaja

**Affiliations:** 1 Department of Molecular and Biomedical Sciences, University of Adelaide, 5005 North Terrace, Adelaide, SA Australia; 2 College of Public Health and Medicine, Molecular Biosciences Theme, Flinders University, Bedford Park, SA 5042 Australia; 3 Organelle Biology and Disease, South Australian Health and Medical Research Institute, Adelaide, SA 5000 Australia; 4 EMBL Australia, Adelaide, SA 5000 Australia; 5 Department of Physiology and Cell Information Systems, McGill University, 3655 Promenade Sir-William-Osler, Montreal, QC H3G 1Y6 Canada; 6 Department of Biochemistry, McGill University, Montreal, QC H3G 1Y6 Canada; 7 Unitat de Fisiologia, Departament de Ciències Fisiològiques, IDIBELL-Institute of Neurosciences, Universitat de Barcelona, L’Hospitalet de Llobregat, Barcelona, Spain; 8 Centro de Investigación en Red de Enfermedades Raras (CIBERER), ISCIII, Madrid, Spain

**Keywords:** Glial biology, Endoplasmic reticulum, Endocytosis, Endoplasmic reticulum, Cell biology, Mechanisms of disease

## Abstract

The significance of crosstalks among constituents of plasma membrane protein clusters/complexes in cellular proteostasis and protein quality control (PQC) remains incompletely understood. Examining the glial (enriched) cell adhesion molecule (CAM), we demonstrate its chaperone-like role in the biosynthetic processing of the megalencephalic leukoencephalopathy with subcortical cyst 1 (MLC1)-heteromeric regulatory membrane protein complex, as well as the function of the GlialCAM/MLC1 signalling complex. We show that in the absence of GlialCAM, newly synthesized MLC1 molecules remain unfolded and are susceptible to polyubiquitination-dependent proteasomal degradation at the endoplasmic reticulum. At the plasma membrane, GlialCAM regulates the diffusional partitioning and endocytic dynamics of cluster members, including the ClC-2 chloride channel and MLC1. Impaired folding and/or expression of GlialCAM or MLC1 in the presence of diseases causing mutations, as well as plasma membrane tethering compromise the functional expression of the cluster, leading to compromised endo-lysosomal organellar identity. In addition, the enlarged endo-lysosomal compartments display accelerated acidification, ubiquitinated cargo-sorting and impaired endosomal recycling. Jointly, these observations indicate an essential and previously unrecognized role for CAM, where GliaCAM functions as a PQC factor for the MLC1 signalling complex biogenesis and possess a permissive role in the membrane dynamic and cargo sorting functions with implications in modulations of receptor signalling.

## Introduction

The crosstalk of membrane protein complexes and cluster members through homo- and heteromerization, protein–protein, protein-lipid interactions, post-translational modifications or cell–cell contacts can regulate complex functional phenotypes^1,2^. Beyond subunit compositions, the biosynthetic secretion efficiency, the plasma membrane (PM) internalization, recycling and degradation kinetics can all influence oligomeric membrane protein steady-state level at the PM^3–6^. Pending on the severity of the conformational defect, genetic mutations can partially or completely disrupt the endoplasmic reticulum (ER) folding/assembly of oligomeric PM proteins and reroute them for premature degradation by specialized organelle protein QC (PQC) mechanisms to eliminate potentially toxic and aggregation-prone proteins^3,7,8^. The significance of crosstalks among constituents of plasma membrane protein clusters/complexes in cellular proteostasis and PQC remains incompletely understood.

GlialCAM (*HepaCAM1*), like the Neural CAM (NCAM), vascular CAM or cluster of differentiation 22 and 48 found in B- and T-cells^9^ mediating cell–cell or cell–matrix interactions and migration, are members of the immunoglobulin (Ig) family CAM. GlialCAM is widely expressed in human cells and abundant in the central nervous system glial cells. In particular, the astrocytic plasma membrane signalling cluster incorporates GlialCAM, the regulatory integral membrane protein MLC1 (megalencephalic leukoencephalopathy with subcortical cysts 1), ClC-2 chloride and TRPV4 (cation) channels, receptors (EGF) and transporters (Na^+^/K^+^-ATPase)^10–14^. This heteromeric cluster has been implicated in the regulation of astrocyte motility, cellular signalling and ion homeostasis^12,15–18^. The expression defect in GlialCAM or MLC1 induces both intracellular and extracellular vacuolization in astrocytes and the brain^11,13,19,20^. Overall, dysfunction of the cluster results in progressive brain defects, difficulties in movement, edema, epilepsy and autism^21–23^.

We have an incomplete understanding of the underlying mechanistic cause of GlialCAM expression defect on the cluster. Intriguingly, loss of expression of MLC1 in the mouse brain causes GlialCAM mislocalization by unknown mechanisms, which in turn perturbs the subcellular distribution of the ClC-2^24^. This implies that GliaCAM (and/or MLC1) modulates through less defined mechanisms direct protein and indirect functional interactions of the cluster members. In support, GlialCAM mutations disrupt MLC1 localization to astrocytic endfeet junctions of blood vessels and cell–cell contacts^11,25–27^ and GlialCAM regulate ClC-2 chloride currents and the volume-regulated anion channel (VRAC/LRRC8) through kinase signalling^12,17^. At least the cellular expression of ClC-2 is not dependent on GlialCAM^11^ in contrast to MLC1, which follows that of GlialCAM. The mice lacking GlialCAM have severely and mutant GlialCAM mice moderately decreased cellular expression of MLC1^24^. This phenomenon could be partly attributed to the ability of GlialCAM to stabilize/localize ClC-2 channel^12,18^ and other members of the cluster with a presently poorly understood molecular mechanism.

Here we uncover that cell adhesion molecule GlialCAM is a PQC factor at multiple cellular locations. We show that GlialCAM expression suppresses the ubiquitin–proteasome system (UPS) mediated ER-associated degradation (ERAD) of MLC1, suggesting that it is required for the biosynthetic conformational stabilization of GlialCAM/MLC1 oligomer. The protection of MLC1 against ERAD was dependent on the cytosolic carboxy (C)-tail of GlialCAM, which was also required for its ER-to-PM targeting and tethering. At the PM, GlialCAM/MLC1 forms a core for the adhesion phase separation module, regulating the endocytosis dynamics of the MLC1, GlialCAM and ClC2 in the cluster. Moreover, we find that lack of GlialCAM or MLC1 provokes fusion stress toward the endo-lysosomal compartment, contributing to the complex molecular brain pathogenesis associated with the cluster. Altogether, these studies highlight the unexpected importance of cell adhesion molecules in PQC for improving the proteostasis health of cells.

## Results

### Destabilization of MLC1 variant at the PM by diseases causing mutations

Megalencephalic leukoencephalopathy with subcortical cysts (MLC), the white matter disease of the brain, is caused by the expression defect of the MLC1 or GlialCAM gene products^11,26^, predominantly confined to the plasma membrane. Molecular mechanisms of how GlialCAM and/or MLC1 expression defects manifest at the PM and subcellular level and modulate each other and the signalling cluster members implicated in a broad range of brain functions are far less understood. As the loss-of-expression phenotype of the mutant MLC1 signalling complex could be similar to a handful of mutant PM proteins attributed to ubiquitination- and ESCRT-dependent lysosomal targeting by the peripheral PQC activity in addition to ER PQC^3,6,28–30^, we assessed first the impact of diseases associated mutations on the PM stabilities of MLC1 variants^31^.

**Figure 1:**
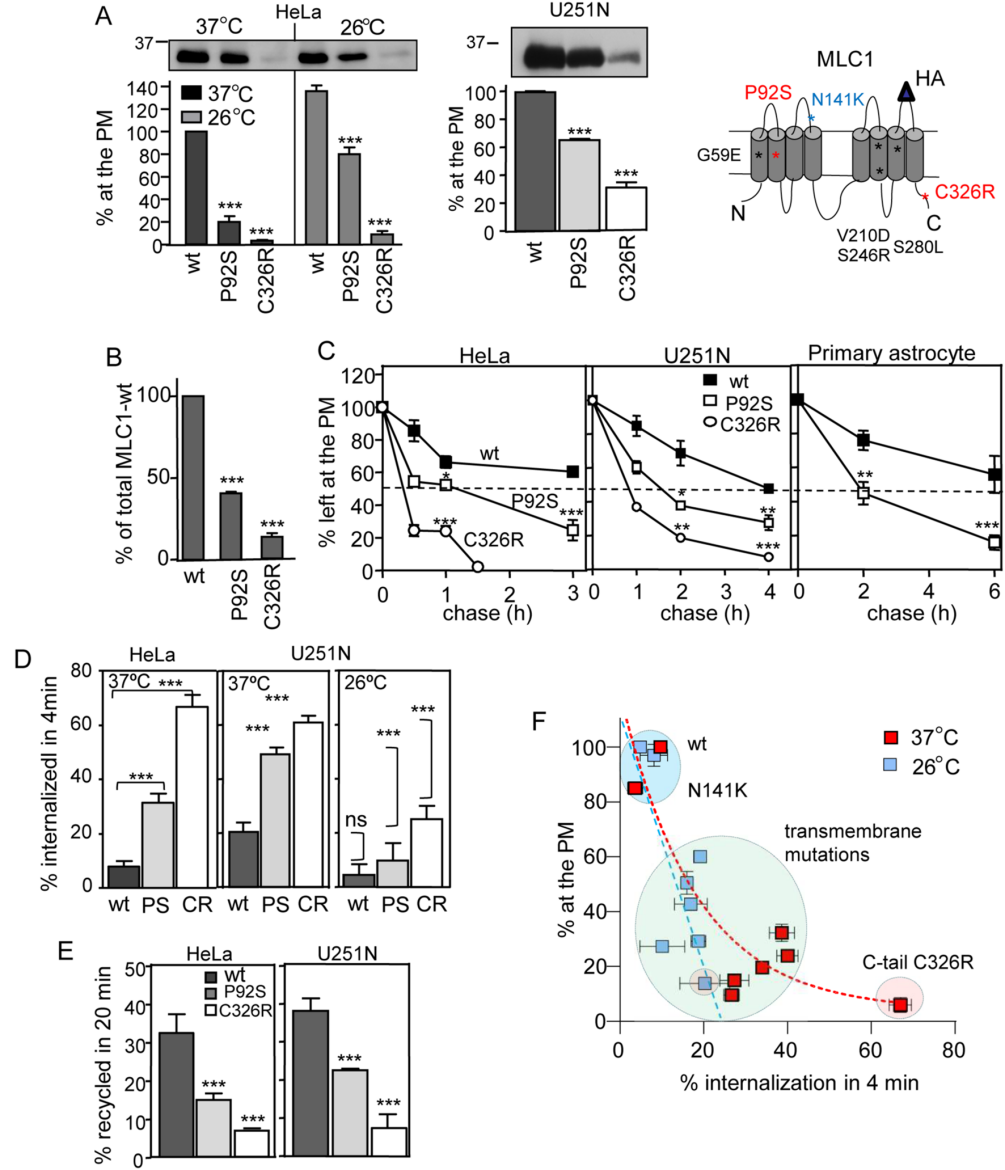
Disease mutations destabilize the regulatory protein MLC1 in multiple cell types. (**A**,**B**) The correlation between the PM density and cellular total protein expression of MLC1 variants (wt, P92S, C326R) was measured at 37 °C and upon temperature rescue (26 °C, 24 h) in HeLa cells by cs-ELISA and Western blot analysis with densitometry. (**C**) The PM turnover of MLC1 variants was monitored in the primary astrocytes, HeLa, and astrocytic U251N cells using cs-ELISA. (**D**) Internalization rates of the MLC1 variants (wt, P92S, C326R) were determined using cs-ELISA. Internalization was initiated at 37 °C for 4 min after anti-HA binding at 4 °C in HeLa cells maintained at 37 °C or after following low-temperature rescue (26 °C, 24 h). Statistical significance in the internalization rate of non-rescued (37 °C) and low-temperature rescued (26 °C) MLC1 variants are indicated on the right panel (U251N, 26 °C). (**E**) The endosomal recycling rates of MLC1 variants [wt, P92S (PS), C326R (CR)] were measured in HeLa and astrocytic U251N cells as described in “Materials and methods”. (**F**) Correlation between internalization rates (%/4 min) and relative cell surface densities (% of the wt) of eight MLC1 variants in HeLa cells at 37 °C (red squares) or after temperature folding rescue at 26 °C (24 h, blue squares). Data are from the Fig. S1B,C. Means ± SEM, n ≥ 3, *p < 0.05, **p < 0.01, ***p < 0.001.

**Figure 2:**
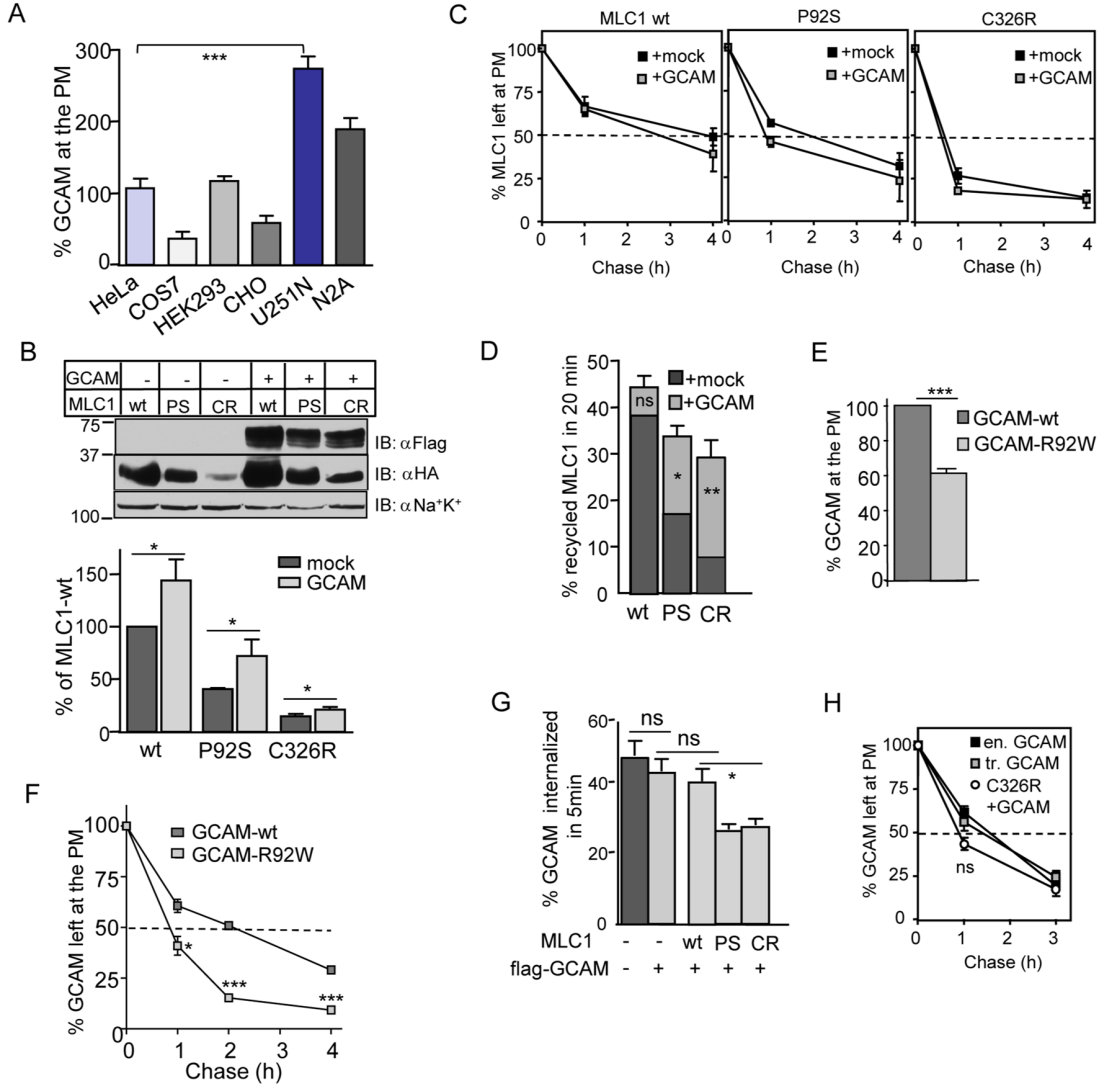
Early endocytic crosstalk of GlialCAM and MLC1 trafficking dynamics. (**A**) The relative PM density of endogenous GlialCAM (GCAM) was measured using cs-ELISA in indicated cell lines and expressed as a percentage normalized for cellular proteins. (**B**) GCAM effect to increase the cellular expression of MLC1 variants (wt, P92S, C326R) was determined by immunoblotting (n = 8). (**C**) The PM stability of MLC1 variants was measured using cs-ELISA in the presence or absence of exogenous GCAM. (**D**) Exogenous GCAM enhances MLC1 variants [wt, P92S (PS), C326R (CR)] endosomal recycling, which was measured using cs-ELISA as described in “Materials and methods”. (**E**,**F**) The PM expression (**E**) and stability (**F**) of GCAM-wt and disease-causing GCAM-R92W were determined using cs-ELISA. GCAM-R92W (~ T_1/2_ < 1 h) was destabilized when compared to its wt counterpart (~ T_1/2_ 2 h). (**G**) The impact of MLC1 variants [wt, P92S (PS), C326R (CR)] expression on the GCAM internalization was measured using cs-ELISA. (**H**) Comparison of the PM stability between overexpressed (tr) GCAM and endogenous GCAM (en) and upon coexpression of MLC1-C326R was measured using cs-ELISA. Means ± SEM, n ≥ 3, *p < 0.05, **p < 0.01, ***p < 0.001.

To interrogate the PM-endosomal PQC contribution to the MLC1 loss-of-expression phenotype, we first determined the cellular and PM expression of the MLC1 variants (Fig. 1A,B) in relation to their PM stabilities (Fig. 1C). The cellular expression was measured using Western blot analysis and the PM expression using extracellular HA-tag in MLC1 and cell surface (cs)-ELISA^28^. GlialCAM (GCAM) expression was adjusted by heterologous expression and siRNA depletion in Hela and astrocytic U251N cells with variable levels of endogenous GlialCAM (see Figs. 1A and 2A).

Both MLC1-P92S and -C326R mutations resulted in a profoundly reduced PM pool (~ 80–90%) (Fig. 1A) and cellular expression (~ 60–80%) (Fig. 1A,B) coinciding with their 3–4-fold increased PM clearance rate relative to the MLC1-wt (Fig. 1C). Both accelerated internalization (Fig. 1D) and defective recycling (Fig. 1E) relative to MLC1-wt account for the fast PM removal of mutants, mediated by specialized peripheral PQC machineries^32^. In astrocytic U251N cells, the MLC1 mutant PM expression was reduced only by 30–70% as compared to HeLa cells, likely due to the higher endogenous GlialCAM expression in U251N cells (Fig. 1A,C, see also Fig. 2A). Importantly, MLC1-wt and P92S turnover were comparable at the PM between primary astrocytes, HeLa and astrocytic U251N cells (Fig. 1C), validating the cell line utility for our subsequent experiments.

Because certain conformationally defective mutant PM proteins could be rescued at low temperature (26 °C)^5,28,29^, we tested whether the PM expression, endosomal recycling and internalization rates of MLC1 disease variants can be correlated to the topological localization of point-mutations (Fig. 1D,E and S1C). The low-temperature exposure resulted in a ~ 4-fold increase in the expression of MLC1-P92S and ~ 2-fold of C326R. Consistent with the conformational rescue of mutants, the MLC1-variants internalization rate was inhibited (Fig. 1D,E and Fig. S1C). As a correlate, thermal unfolding at 40 °C (2 h) further accelerated internalization rates of P92S and C326R by 2.5-fold, conceivably due to enhanced unfolding propensity of mutants at 40 °C following their temperature rescue (Fig. S1A).

Comparable PM expression defect was rendered by five missense mutations in transmembrane regions of MLC1, causing ~ 2–6-fold increased internalization rate and low-temperature folding rescue of the MLC1-wt (Fig. 1F, Fig. S1B,C). The only outlier was the N141K mutation in the second extracellular loop, displaying a wt-like expression pattern, suggesting that some of the ~ 80 diseases causing MLC1 mutations^31^ may exhibit solely a functional defect without apparent conformational impairment. On the other hand, the ~ 3–4-fold reduced PM expression of MLC1-S246R and -S280L, could be attributed, at least partly, to their ~ 4-fold enhanced internalization rate (Fig. 1F). In contrast, increased internalization (~ 2.5 fold) jointly with reduced ER processing efficiency can explain the ~ 6–10-fold loss of MLC1-G59E and V210D PM expression. These variants represent a class of mutations that are recognized and eliminated by the combined activities of the ER and PM-endosomal PQC similar to hERG long QT heart syndrome PAS-domain mutations^6^. Thus MLC1 disease-causing point mutations can confer folding and peripheral stability defects that additively diminish signalling cluster density at the PM.

### Both GlialCAM and MLC1 modulate endosomal trafficking dynamics of the heteromer

**Figure 3:**
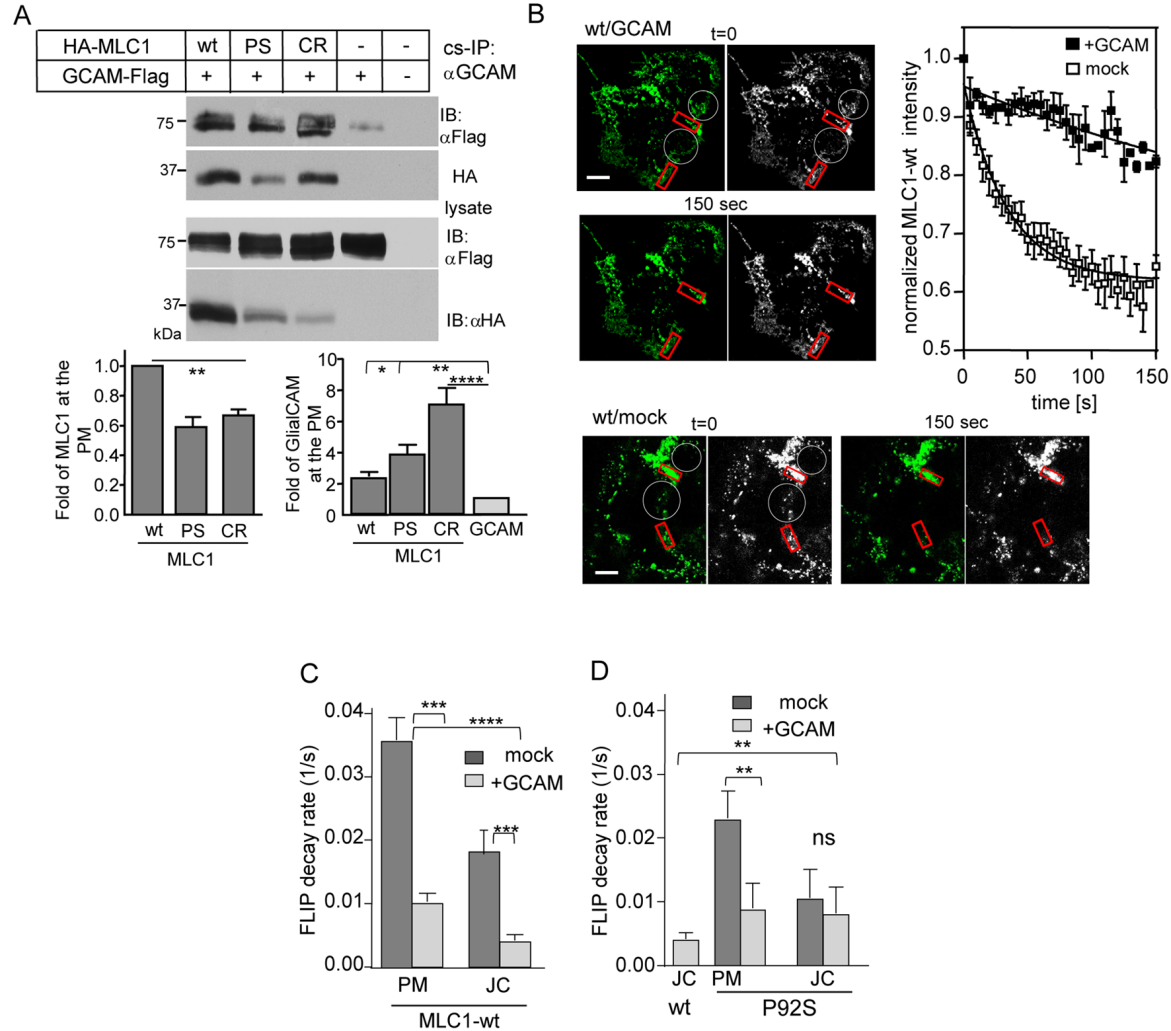
GlialCAM is a diffusional phase barrier for the MLC1 signalling cluster at the PM. (**A**) The indicated HA-MLC1 variants (wt, P92S (PS), C326R CR) interacting with GlialCAM (GCAM) were isolated from the PM using cs-IP with anti-GCAM Ab. The precipitates were analyzed using Western blot analysis with anti-HA or anti-Flag Abs. MLC1 was measured as fold change to wt (left panel) from co-IP-ed samples and the amount of GCAM pulldown was normalized to sample lacking MLC1 (right panel) in HeLa cells. (**B**) MLC1 lateral diffusion at the PM was measured by FLIP. Indicated areas (white circles) were photobleached and the fluorescence loss was monitored in the adjacent red squares. The normalized intensity traces show fluorescence decay in cells +**/−** GCAM overexpression. (**C**,**D**) Summary of decay rate constants are expressed as 1/s for MLC1-wt (**C**) and P92S (**D**) in cells +**/−** GCAM overexpression. GCAM substantially restricts the diffusion of MLC1-wt at the free PM (PM) and cell–cell junctions (JC), in contrast to P92S showing restriction only at the free PM. Statistical analysis against mock (J-D) or wt in the junction (D) are indicated. Means ± SEM, n ≥ 3, **p < 0.01, ***p < 0.001, ****p < 0.0001.

Because molecular pathogenesis of GlialCAM mutations and their folding, targeting and/or oligomerization can impact the cellular fate of MLC1, we examined the impact of GlialCAM on the PM-endosomal stability of the MLC1. The ~ 3-fold lower endogenous GlialCAM expression of Hela than astrocytic U251N cells at the PM (Fig. 2A, see also Fig. S5E) fostered the modulation of GlialCAM levels in Hela. Cellular expression was measured using Western blot analysis and the PM expression using cs-ELISA. Overexpression of GlialCAM-Flag increased the cellular expression of MLC1-wt, -P92S and -C326R, but elevated only the MLC1-C326R PM density (Fig. 2B, Fig. S2A, see also Fig. 3A). GlialCAM overexpression marginally altered the PM stability and internalization of MLC1 variants (Fig. 2C, Fig. S2B). This suggests that the endogenous adhesion molecule expression is sufficient to support MLC1-wt expression into the cluster. Nevertheless, GlialCAM accelerated the endosomal recycling of MLC1-P92S and C326R without impact on wt (Fig. 2D). Similarly, severe MLC1-C326R increased the PM density and endosomal recycling of GlialCAM (Fig. S2C,D).

The disease-causing R92W mutation in the N-terminal extracellular IgV-domain of GlialCAM^18^ reduced the PM expression and stability of GlialCAM (Fig. 2E,F), reminiscent of the MLC1 mutants cellular phenotype. In contrast, the expression of MLC1-P92S or C326R significantly impeded the internalization rate of GlialCAM (Fig. 2G), which led to a marginal change in the PM stability of GlialCAM (Fig. 2H). Importantly, the PM stabilities of endogenous and overexpressed GlialCAM were comparable (Fig. 2H). Jointly, these results suggest that GlialCAM cannot suppress the accelerated internalization of MLC1 disease variants and ~ 3-fold faster PM turnover persisted upon GlialCAM overexpression (Fig. 2C).

### GlialCAM regulates the lateral PM mobility of MLC1

Coupled endosomal recycling dynamics between GlialCAM and MLC1 (Figs. 1E, 2D) implies that GlialCAM expression level may determine the membrane phase-separation of the PM signalling cluster, acknowledged as a regulator of the transmembrane adhesion, receptor signalling, and cell junction formation^1,33,34^. To examine the physical proximity and expression level effect of GlialCAM on MLC1 at the PM, we performed cs-co-IP using an antibody against the extracellular epitope of GlialCAM. GlialCAM interacted with all MLC1 variants at the PM (Fig. 3A). The PM density of GlialCAM was increased by ~ 2–7-fold upon MLC1-wt, P92S, or C326R expression, as compared to that without MLC1, in line with the PM density measurements of GlialCAM (Fig. 3A; lower right panel, Fig. S2C). Consistent with the ~ 3-fold enhanced recycling of GlialCAM in MLC1-C326R expressed cells (Fig. 2D), it provoked the largest GlialCAM increase in the IP.

In principle, GlialCAM could enhance extracellular, cell–cell junctional and/or cytoskeletal tethering resulting in reduced diffusional mobility of the cluster. To better understand the impact of GlialCAM expression on the MLC1 lateral diffusional mobility, we performed fluorescence loss in the photobleaching (FLIP) measurement on the extracellularly labelled PM resident MLC1 variants. GlialCAM overexpression (as in Fig. S2C) profoundly delayed the lateral mobility of MLC1 variants at non-junctional PM regions (Fig. 3B–D, Fig. S3A,B), suggesting that an increase in GlialCAM intracellular interactions and homo-oligomerization is sufficient to enhance the tethering. Further, increased GlialCAM tethering by both intra- and extracellular interactions at junctional regions (JC in figures) was proportional to MLC1 variant folding state, where GlialCAM reduced mobility rates of MLC1-wt by ~ 47%, P92S ~ 60%, and C326R ~ 85% relative to endogenous GlialCAM control (Fig. 3C,D, Fig. S3C). This is likely due to cell junctions forming a stronger diffusional barrier^35^ and MLC1 variants proportional expression dependency of GlialCAM observed in Figs. 2 and 3A. Moreover, the lateral mobility rates of MLC1-wt, -P92S and -C326R were similar both in presence of endogenous or overexpressed GlialCAM at non-junctional regions, implying that despite the attempts to maintain the cluster stoichiometry, the tethering itself is largely insensitive to the MLC1 conformational state (Fig. S3D). Thus, both GlialCAM and MLC1 expression levels can influence the overall tethering and mobility of the cluster at the PM.

### GlialCAM/MLC1 regulates endosomal dynamics of the signalling cluster

**Figure 4:**
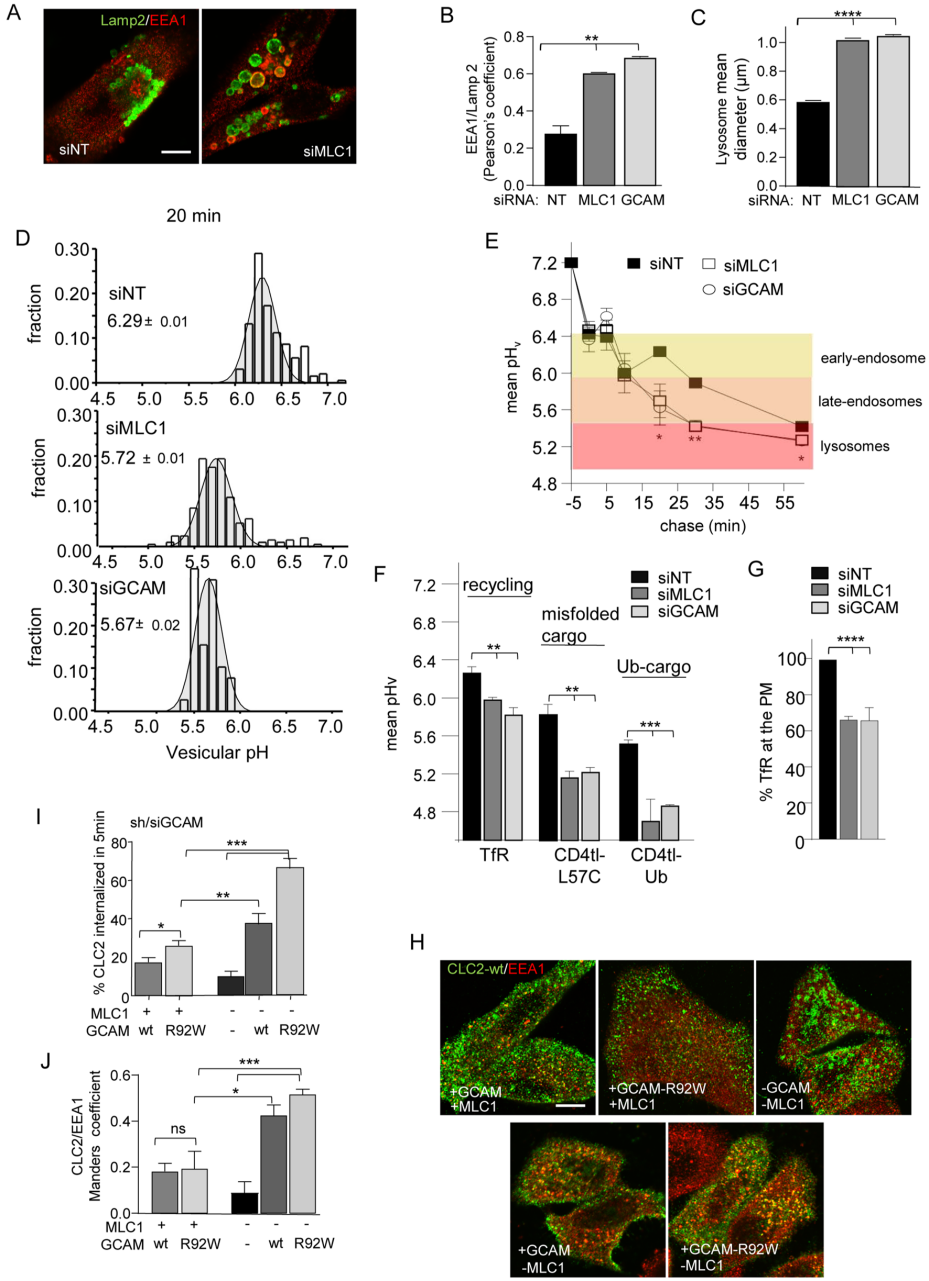
A misaligned GlialCAM signalling cluster causes late endosomal enlargement and increases the early endosomal resident time of ClC-2. (**A**) MLC1 expression effect on late endosomes-lysosomes was monitored using siMLC1 and siNT (non-targeted) mediated depletion in astrocytic U251N cells. Lamp2 marker is for lysosomes and EEA1 for early endosomes. Bar: 5 µm. (**B**) High-content image analysis of immunostaining and treatments in panel (**A**). EAA1/Lamp2 + colocalization was analyzed using Pearson’s correlation coefficient. siNT was used as a control in comparison to siMLC1 and siGCAM depleted U251N cells (~ 300/repeat, n = 3). (**C**) As in (**B**), but the mean diameter of individual Lamp2^+^ lysosomes was measured (n = 21,656–47,877/repeat of lysosomes). (**D**) Representative histograms of the endo-lysosomal pathway vesicular pH (pHv) measurement in siNT, siMLC1 and siGCAM astrocytic U251N cells. Live cells were loaded with pH-sensitive dextran for 5 min and chased for 20 min before single vesicle analysis. (**E**) Mean vesicular pH (pHv) of cells in (**D**) chased for indicated times. Characteristic endosomal pH in control siNT cells is indicated. (**F**) Mean vesicular pH (pHv) of cells in (**D**) labelled for transferrin receptor (TfR) containing recycling or misfolded (CD4tl-L57C) and constitutively ubiquitinated (CD4tl-Ub) model cargo endosomes. (**G**) Strongly recycling transferrin receptor (TfR) amount at the PM was measured using cs-ELISA. (**H**) Immunofluorescence of internalized ClC2 and colocalization with EEA1 in cells +**/−** GCAM and +**/−** MLC1 overexpression in HeLa cells. Bar: 5 µm. (**I**) The impact of GCAM-wt, GCAM-R92W, and MLC1 expression on chloride channel HA-ClC-2 internalization was measured for 5 min at 37 °C using cs-ELISA in HeLa cells. (**J**) Manders' overlap coefficient of ClC2 with EEA1 + early endosomes. Means ± SEM, n ≥ 3. p-value: *ns* non-significant, *p < 0.05, **p < 0.01, ***p < 0.001 ****p < 0.0001.

The precise function of MLC1 remains enigmatic, but as a scaffolding molecule, it may exert its regulatory functions through multiple members of the GlialCAM/MLC1 signalling cluster. To gain more insights into the MLC1 heteromeric signalling complex function, we established morphological and functional changes at the endo-lysosomal compartment upon depletion of endogenous MLC1 or GlialCAM. Direct or indirect depletion of MLC1 by siMLC1 or siGlialCAM, respectively, resulted in a significantly enlarged endosomal compartment that could be, at least partly, attributed to impaired maintenance of the identity of endocytic organelles, reflected by the overlapping colocalization of markers of early endosomes (early endosomal antigen1 (EEA1)) with multivesicular bodies/lysosomes (Lamp2^+^) (Fig. 4A–C, Fig. S4A,B). High-content image analysis confirmed that the Pearson colocalization coefficient of EEA1 with the lysosomal marker Lamp2 increased by more than ~ 2-fold from 0.27 ± 0.04 in non-targeted siRNA (siNT) exposed cells to 0.68 ± 0.004 or 0.61 ± 0.002, (n = 3; ~ 300 cells/repeat) in MLC1- or GlialCAM-depleted cells, respectively (Fig. 4B). This was accompanied by a similar reduction in the number of lysosomes by ~ 43% from 154 ± /12 in siNT to ~ 68 ± 16 in siMLC1/GCAM cells (Fig. S4C) exclusively stained for Lamp2. Concomitantly, the mean diameter of the siNT treated lysosomes (0.58 ± 0.001 µm) increased ~ 56% when MLC1 (~ 1.02 ± 0.003 µm) and GlialCAM (~ 1.05 ± 0.003 µm) was depleted (Fig. 4C). Jointly, these observations suggest that MLC1 haploinsufficiency severely perturbs the identity of endo-lysosomal organelles, indicative of their homeostatic/proteostatic stress^36^.

Next, the functional consequences of the endo-lysosomal homeostatic perturbations were examined by monitoring compartment-specific regulation of endo-lysosomal pH. The gradual acidification is a hallmark of the endo-lysosomal compartment, starting at near-neutral pH of endocytic vesicles and progressing via early- (pH 6–6.4) and late-endosomes/MVB (pH 5.5–6.0) to the highly acidic lysosomes (pH < 5.5)^5,28,37–39^. To determine whether the enlarged early-late-endosomal compartment is connected to altered acidification, we used pH-sensitive single vesicle fluorescence microscopic analysis in live cells^5,28,37,38^. Following the fluid-phase labelling of the endosomal compartments with the pH-sensitive dextran with a 5 min pulse, the luminal pH of the endocytic compartment was measured as a function of chase time (2–60 min, 37 °C)^37,38^. Both GlialCAM- and MLC1-depletion resulted in the accelerated acidification of the dextran-labelled compartment(s) after 20 min chase, likely due to the increased propensity of the early/late endosomes and lysosomal fusion or impeded fluid-phase exocytosis (Fig. 4D,E).

We also examined the intracellular itinerary of several cargo molecules with distinct sorting signals. The pH of TfR receptor-labelled recycling endosomes was decreased in MLC1 (5.97 ± 0.03) and GlialCAM (5.82 ± 0.08) depleted cells compared to non-targeted siRNA treated cells (siNT; pH 6.26 ± 0.07) (Fig. 4F). Both the misfolded chimeric transmembrane model protein (CD4tl-L57C) and the constitutively ubiquitinated (CD4tl-Ub) model cargoes, which undergo ubiquitin- and ESCRT-mediated cargo sorting toward lysosomes^28^, were confined to significantly lower pH compartment in MLC1- or GlialCAM-depleted than in control cells after 1 h chase indicating accelerated lysosomal sorting and/or fusion and/or hyper-acidification of late-endosomes (Fig. 4F). While the precise mechanism of the altered organellar pH regulation remains to be elucidated, these unexpected results suggest a fundamentally altered pH homeostasis and consequential cargo sorting recycling/kinetics along with the endo-lysosomal compartments. Confirming this, the amount of TfR recycling was decreased by ~ 35% at the PM (Fig. 4G), similarly to the uptake and recycling of the horseradish peroxidase (HRP), a fluid-phase marker, by > 30% (Fig. S4D) and ~ 10%, respectively (Fig. S4E). Importantly, MLC1-S280L was not able to rescue HRP trafficking defects (Fig. S4D,E). These results show that despite enlargement, the endo-lysosomal compartment acidification is preserved, but the loss of compartmentalization severely perturbs endosomal cargo sorting and recycling, an indication of the endosomal compartment stress.

### Permissive role of GlialCAM/MLC1 on the regulation of ClC-2 at the PM-endosomes

As the expression of GlialCAM and MLC mutually influences their PM partitioning, tethering (Fig. 3, Fig. S3), and endocytosis dynamics (Fig. 2, Fig. S2), we posit that GlialCAM may form a bridge between ClC-2 to MLC1 in the ternary complex and a similar paradigm of membrane partitioning may prevail for ClC-2. To examine this scenario, we determined the ClC-2 channel PM density and residence time in early endosomes in cells depleted for GlialCAM or expressing GlialCAM disease mutant. Immunofluorescence microscopy showed that ClC-2 clustered to structures outside the ER in cells lacking MLC1, especially when GlialCAM was depleted or mutated (R92W) (Fig. 4H, Fig. S4F,G). Cs-ELISA and colocalization assays uncovered that in the absence of MLC1, GlialCAM-wt and -R92W significantly increased internalization and colocalization of ClC-2 with EEA + early endosomes (Fig. 4H–J). In contrast, the lack of both GlialCAM and impeded MLC1 internalization and promoted the accumulation of isolated clusters of ClC2 at the PM and, possibly at the sub-plasma membrane regions in the form of budding endocytic vesicles (Fig. 4H, see -GCAM/-MLC). Whereas GlialCAM-R92W rendered faster internalization of the ClC2 than GlialCAM-wt in the presence of MLC1 (Fig. 4J), we could not detect a significant difference in endosomal localization (Fig. 4I). Taken together, the analysis of GlialCAM provided insights into the compositional partitioning and its effect on the functionality of the PM-endosomal compartment.

### GlialCAM stabilizes the MLC1-complex at the ER

We have shown that GlialCAM ablation in primary astrocytes or knockout mice abolished the junctional and cell surface localization, and reduced the total expression of MLC1^11,24,40^. While this phenotype is consistent with GlialCAM regulating the targeting of MLC1, the underlying mechanism of the severe expression defect of MLC1 caused by the loss-of-GlialCAM expression remains largely speculative. Here we showed that GlialCAM overexpression significantly elevated the steady-state cellular expression of MLC1 without influencing its peripheral metabolic turnover (Fig. 2B). We envision three possible mechanisms of actions of GlialCAM on MLC1 biogenesis. (i, ii) GlialCAM association may mask the ER retention signal (i) or expose ER export signals (ii) identified in the MLC1 cytosolic segments^10^, or (iii) GlialCAM binding allosterically stabilizes and/or promote the folding of the MLC1 in the heteromeric complex. Notably, the efficacies of these functional modalities of GlialCAM may also be influenced by MLC1 mutation-induced conformational changes.

**Figure 5:**
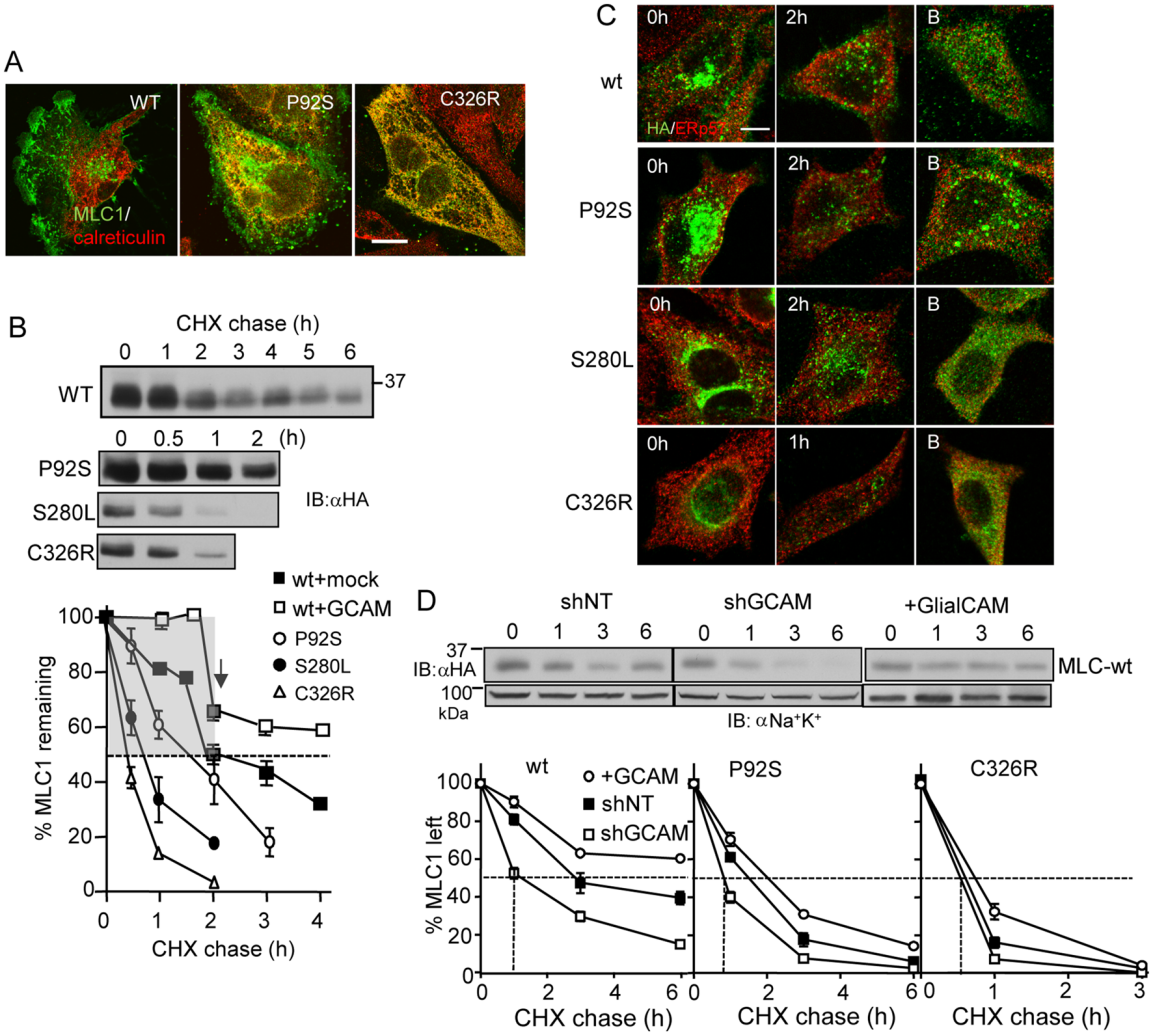
GlialCAM increases the ER stability of MLC1. (**A**) Immunofluorescence co-localization of MLC1 variants with the ER marker calreticulin. MLC1 variants were detected with indirect immunostaining using an anti-HA primary antibody in astrocytic U251N. Notably, the MLC1-wt displays marginal accumulation in the ER. Bar 5 µm. (**B**) Cellular turnover of MLC1 variants was monitored upon translational inhibition with cycloheximide (CHX) for 1–6 h. Western blot analysis revealed the fast (gray area) ER-clearance and the slow post-ER pools of MLC1-wt during CHX-chase in comparison to only the fast turnover pools of disease associated mutants. (**C**) Immunostaining of MLC1 variants before and after CHX chase (1–2 h) in the absence or presence of proteasomal inhibition (**B**) with Bortezomid (1 µM) during the CHX treatment. MLC1 was detected with an anti-HA antibody and ERp57 was used as an ER marker. Bar 5 µm. (**D**) The GCAM effect on the total cellular turnover of MLC1 variants was measured by immunoblotting during CHX-chase in HeLa cells. GCAM was overexpressed or depleted ~ 60% with a short hairpin (sh). Non-target shRNA (shNT) was as a control. Non-tr; non-transfected, lys; lysate. Means ± SEM, n ≥ 3.

First, we established the subcellular distribution and turnover of MLC1-HA variants in the calreticulin-labelled ER by using the immunofluorescence co-localization technique. MLC1-wt was primarily confined to the PM adhesion processes in astrocytic U251N cells and was absent from the ER (Fig. 5A, Fig. S5A). In contrast, the mild P92S and the severe C326R mutation displayed a partial and near-complete colocalization, respectively, with calreticulin (Fig. 5A, Fig. S5A). The cellular turnover of MLC1 variants was monitored using immunoblotting after translational termination with cycloheximide (CHX). MLC1-wt exhibited biphasic degradation kinetics with fast (T_1/2_ < 2 h) and slower turnover pools (T_1/2_ > 2 h) (Fig. 5B). In contrast, MLC1 mutants, predominantly confined to the ER, displayed a monophasic decay with T_1/2_ of ~ 1 h for S280L, ~ 0.5 h for C326R and ~ 1.5 h for P92S (Fig. 5B).

We postulated that a fastly turning over pool of MLC1 represents the proteolytic degradation of newly synthesized MLC1 nascent chains or conformationally defective heteromeric complexes, and are therefore subjected to recognition and elimination by the ubiquitin–proteasome system, a constituent of the ER PQC^8^. Supporting this hypothesis, the CHX-induced disappearance of mutants was delayed with the proteasome inhibitor Bortezomib (Fig. 5C, Fig. S5C). Conversely, MLC1-wt and the mild disease P92S mutation exhibited slower cellular turnover that could be attributed to more efficient conformational maturation and/or cluster assembly in the presence of endogenous GlialCAM. The steady-state accumulation of these variants was promoted by their slower degradation in post-Golgi compartments (Fig. 5B,C, Fig. S5B).

The ER confinement with efficient and near-complete clearance of the MLC1-S280L and -C326R cellular pools argue against the possibility that GlialCAM could selectively mask an ER-export motif(s) or induce the exposure of ER-retention signal(s) in these MLC1 variants. Both scenarios would manifest accumulation in the ER of the transport-incompetent but natively folded mutants as described for other membrane proteins^8,39^, however, this was not the case for MLC1 (Fig. 5D).

To directly assess the role of GlialCAM in the MLC1-wt biogenesis, a short hairpin (sh)RNA depletion was established, a prerequisite to generate loss-of-function and gain-of-function phenotype for GlialCAM (Fig. 5D, Fig. S5D–F). Depletion of GlialCAM further accelerated the turnover of MLC1-wt and P92S pools to T_1/2_ < 0.5 h (Fig. 5D). Conversely, GlialCAM overexpression delayed both the fast phase of MLC1-wt turnover to T_1/2_ ~ 2 h and the second slower to ~ 6 h (Fig. 5D). A similar trend was observed for P92S turnover, but without a significant impact on C326R, displaying the fastest metabolic turnover in the ER (Fig. 5D, Fig. S5D). Part of the C326R and to a lesser extend of P92S turnover was also catalyzed by lysosomal degradation since inhibition of the autophagosomal-lysosomal proteolysis of lysosomal cathepsins with leupeptin (L) or formation of autophagosomes with 3-methyladenine (A) delayed the mutants metabolic turnover (Fig. S4A). Jointly, these observations support our conclusion that GlialCAM is not merely a permissive factor for MLC1 secretion from the ER but it is essential for the conformational-dependent metabolic stabilization of both the wt and some of the mutant MLC1 variants as well at the ER.

### GlialCAM promotes MLC1 conformational maturation at the ER

**Figure 6:**
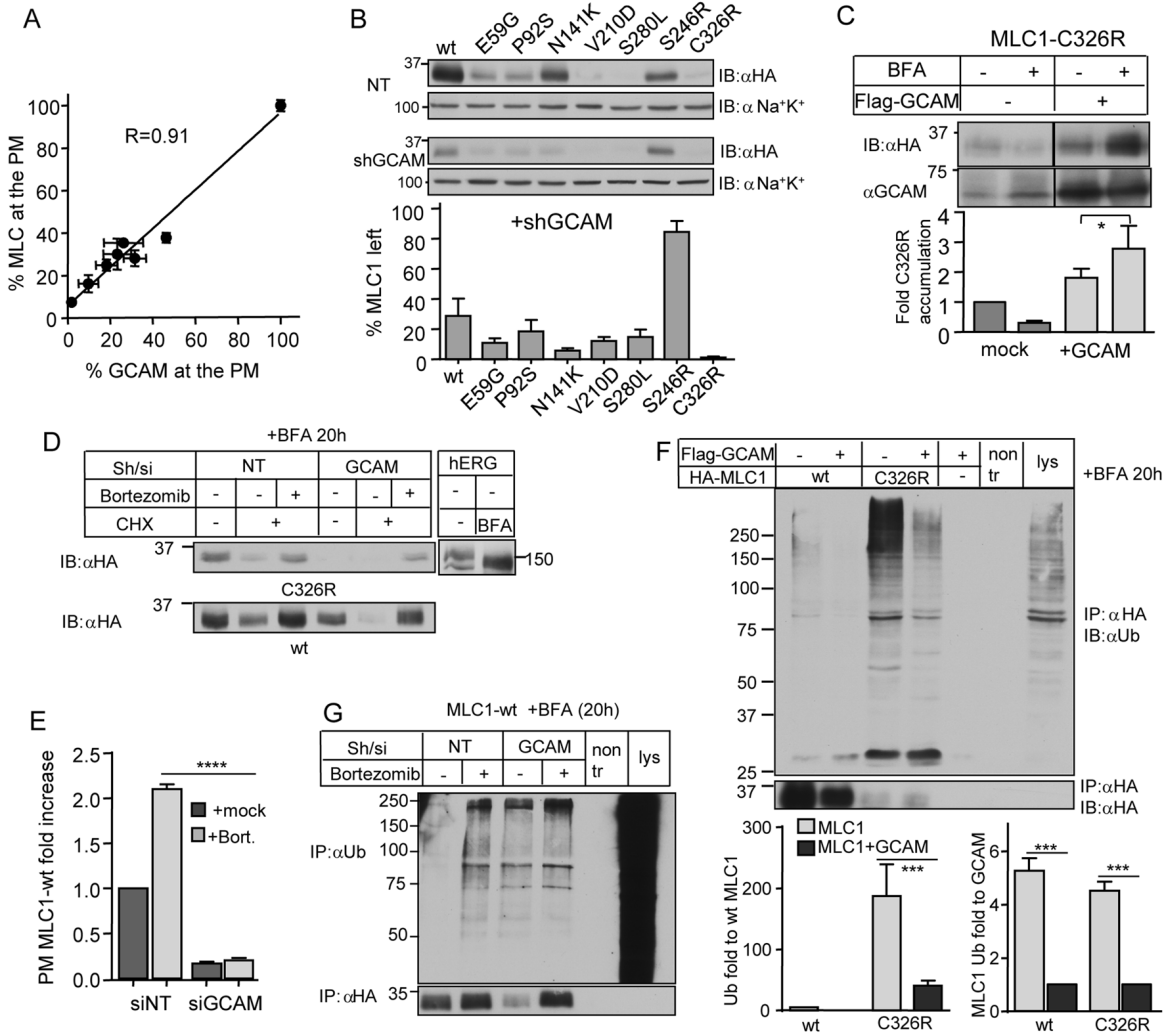
The cell adhesion protein, GlialCAM, protects MLC1 against ubiquitination and degradation at the ER. (**A**) The correlation of the PM expression between endogenous GlialCAM (GCAM) and exogenous MLC1-wt was measured using cs-ELISA in HeLa cells and expressed as a percentage of mock-treated MLC1-wt cells. The GCAM downregulation efficiency was altered by using different amounts of siGCAM. (**B**) Western blot analysis of GCAM downregulation on the cellular expression of MLC1 variants was determined after depleting ~ 60% of GCAM with short hairpin (sh)RNA or shNT (non-targeted). Total MLC1 expression was quantified against the loading control Na^+^–K^+^-ATPase (Na^+^K^+^). (**C**) GlialCAM overexpression protects MLC1-C326R against rapid ER elimination. Immunoblot analysis of MLC1 and GCAM expression levels was carried out after 20 h BFA (5 µg/ml) or mock-treated HeLa cells with or without GlialCAM overexpression. (**D**) The ER-confined MLC1-wt and C326R degradation were monitored in fully GCAM (sh/si) depleted or control (NT) HeLa cells. The ER-to-Golgi transport was inhibited with BFA (5 µg/ml) for 20 h, and the ER was cleared using CHX-chase for 30 min with or without proteasomal inhibition (Bortezomib, 1 µM). The absence of complex glycosylation in the hERG channel confirmed BFA efficacy by disrupting ER-to-Golgi transport. (**E**) The influence of GCAM expression on the PM amount of MLC1-wt was measured using cs-ELISA. Proteasomal inhibition (Bortezomib, 2 h) was unable to rescue the PM expression of MLC1 when GCAM was partially depleted as in panel Fig. 4B. (**F**) GCAM overexpression effect on the MLC1 ubiquitination at the ER was measured in HeLa cells treated with BFA for 20 h and +**/−** GCAM. MLC1-wt or C326R were IPed with anti-HA under denaturing conditions. The ubiquitin signal was normalized to MLC1 expression in the IP (left panel). GCAM overexpression decreases the normalized MLC1-C326R ubiquitination by ~ 5-fold (right panel). (**G**) The overexpression effect of GCAM on MLC1-wt ubiquitination at the ER was measured in cells treated with BFA for 20 h. GCAM was depleted (si/sh) or cells treated with non-target (NT) and proteasomal inhibitor (Bortezomib, 2 h). MLC1-wt was immunoprecipitated with anti-HA under denaturing conditions to measure direct ubiquitination. Means ± SEM, n ≥ 3. ****p < 0.0001.

To further evaluate whether GlialCAM is an essential or only facultative folding stabilizer of MLC1, we progressively downregulated GlialCAM using different amounts of siRNAs (Fig. S5E) and in parallel monitored the expression of both MLC1-wt and GlialCAM at the PM. Our results unravelled a linear correlation between the GlialCAM and MLC1 expression at the PM (Fig. 6A). Notably, near-complete depletion of GlialCAM reduced the PM expression of MLC1-wt by > 95% (Fig. 6A). Western blot analysis showed that > 80% downregulation in endogenous GlialCAM elicited > 90% loss of cellular expression of disease MLC1 variants (except the S246R) (Fig. 6B). Thus, GlialCAM is indispensable for folding/conformational stabilization and escape of newly synthesized MLC1-wt molecules in the heteromeric signalling complex from the ER QC.

To reinforce our conformational stabilization model, we determined the fate of newly synthesized MLC1 molecules in the ER after inhibition of the ER-to-Golgi vesicular transport with Brefeldin A (BFA). It is worthy of notice that natively folded membrane proteins are resistant to the ER QC and their stability in the ER and post-ER compartments are comparable^41^. GlialCAM overexpression was increased the accumulation of ER-retained MLC1-C326R by ~ 3-fold, conceivably by partially suppressing its conformational defect (Fig. 6C). In accordance, depletion of the endogenous GlialCAM prevented the ER accumulation of both MLC1-wt and C326R, which was reversed by proteasomal inhibition (Fig. 6D). Importantly, the PM expression of MLC1-wt was enhanced by proteasomal inhibition only when GlialCAM was expressed, but not in its absence (Fig. 6E). Jointly, these results provide support for our model that GlialCAM loss-of-function/expression severely compromises the conformational maturation of MLC1 variants in the heteromeric complex, which becomes susceptible to the ER retrotranslocation and proteasome-dependent degradation. Consistently, dominant GlialCAM mutations also cause a defect in MLC1 expression^20^, as observed in GlialCAM-knockdown mice^24^.

### GlialCAM protects MLC1 from ubiquitination in the ER

Ubiquitination represents and a well-established targeting signal of conformationally defective membrane proteins for the ERAD by the UPS^42^. The effect of GlialCAM on the ER-trapped MLC1 ubiquitination levels was determined using denaturing immunoprecipitation (IP) to preclude isolation of interacting ubiquitinated proteins followed by anti-ubiquitin (Ub) Western blot analysis. GlialCAM overexpression reduced ~ 5-fold both MLC1-wt and C326R polyubiquitination (Fig. 6F). Under the same condition, GlialCAM was moderately ubiquitinated (Fig. S6). Importantly, depletion of GlialCAM led to a profound increase in polyubiquitination of MLC-wt at a comparable to that observed upon proteasomal inhibition (Fig. 6G). This gives further credence to our conformational model showing that GlialCAM interacts at the ER with both unassembled and/or folding intermediates of MLC1-wt and misfolded C326R. Therefore, we suggest that the chaperone-like function of GlialCAM can limit the ERAD susceptibility of MLC1 by shifting the hetero-oligomeric MLC1 complex toward its native-like conformation.

### The C-terminal tail of GlialCAM rescues the MLC1 expression defect

**Figure 7:**
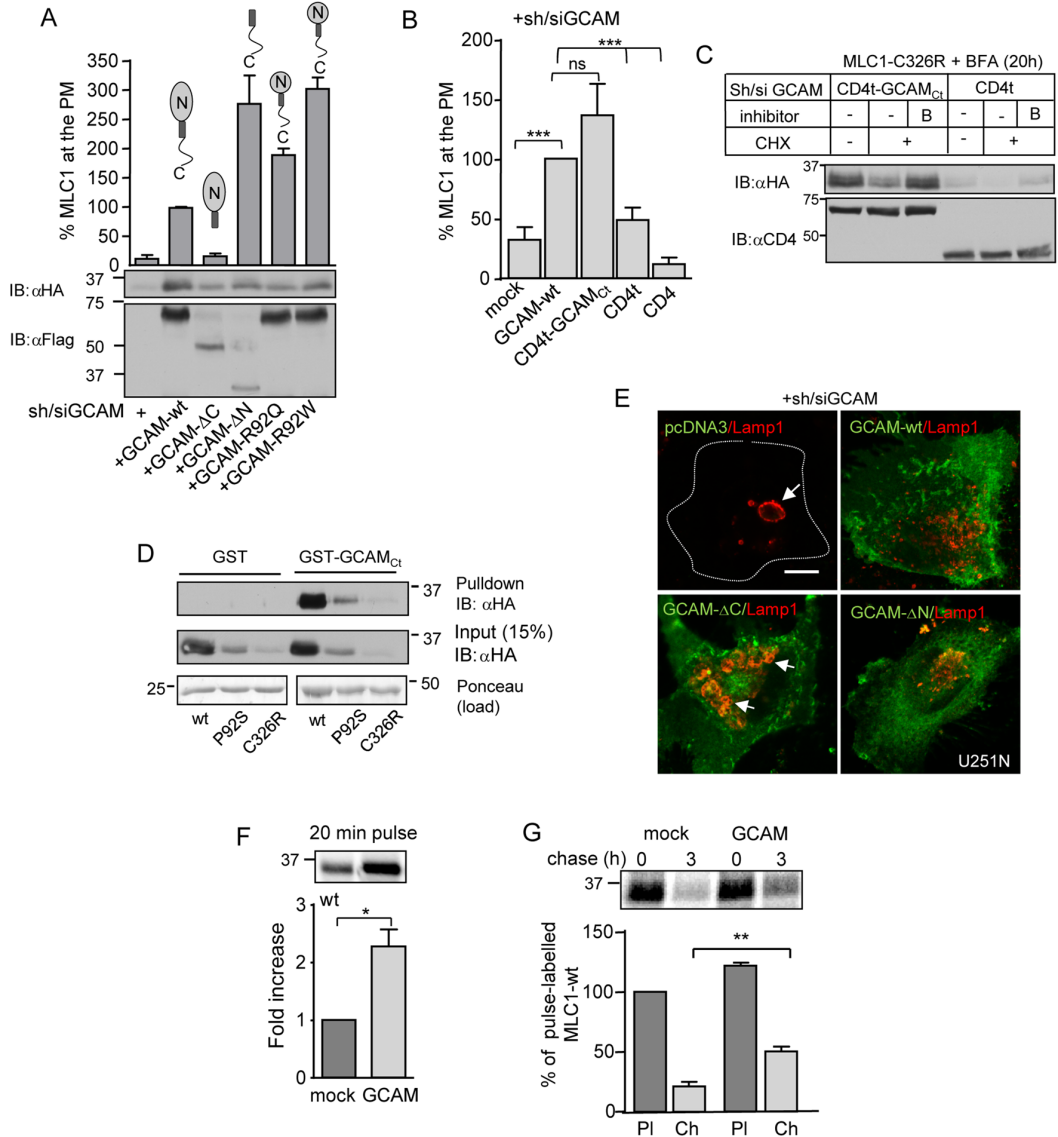
GlialCAM is a chaperone-like folding assistant at the ER to enhance MLC1 biosynthetic secretion. (**A**) Structural requirements of GlialCAM (GCAM) for MLC1 trafficking to the PM. The PM density of MLC1 was measured using cs-ELISA in GCAM-depleted (si/sh) HeLa cells and expressing C- or N-terminally truncated GCAM-Flag variants as indicated. Western blot analysis is shown in lower panels. The C-terminal tail was minimally required to enable the PM targeting of MLC1. GCAM-R92Q or W are MLC disease-causing mutations. (**B**) The effect of the C-terminal tail of GCAM in trans on the PM expression of MLC1 was measured using cs-ELISA. Cells were depleted for GCAM (si/sh) and coexpressed with the C-terminal tail of GCAM fused to the truncated CD4, lacking its cytoplasmic tail (CD4t-GCAM_Ct_). CD4t and CD4 were used as controls. (**C**) Western blot analysis of GCAM_Ct_ effect on the ER-retained MLC1 in the presence of BFA was performed as in panel (**D**), except using CD4t-GCAM_Ct_ coexpression and CD4t as a control. (**D**) Pulldown of MLC1 variants from cell lysates with the GST or GST fusion protein, containing the GCAM_Ct_ (GST-GCAM_Ct_). (**E**) The effect of GCAM truncation on the endo-lysosomal pathway morphology was monitored in GCAM depleted (si/sh) astrocytic U251N cells. GCAM expression was detected with anti-GCAM and lysosomes with anti-Lamp1 Ab. Arrow points to enlarged Lamp1 + lysosomes. Bar 5 µm. (**F**) Phosphorimage analysis of metabolic pulse labelled (20 min, 37 °C) MLC1 with [^35^S] methionine/cysteine in +**/−** of GlialCAM overexpressing cells. (**G**) GlialCAM effect on the maturation of MLC1. Metabolic pulse-chase experiments as in panel (**E**), but using 1 h pulse-labelling at 26 °C and 3 h chase at 37 °C to allow the complete degradation of the ER-resident labelled MLC1 pool. Means ± SEM, n ≥ 3. *p < 0.05, **p < 0.01, ***p < 0.001.

To identify the GlialCAM domain responsible for interaction with MLC1 in the ER, we deleted the N-terminus (ΔN) and C-terminus (ΔC) tails, as well as inserted two disease-causing point-mutations (R92Q, R92W) into the N-terminal domain of GlialCAM^18^, exposed to the ER-lumen, characteristic for a type I transmembrane proteins^18^. GlialCAM variants were expressed in cells depleted for endogenous GlialCAM and expression was confirmed by Western blot analysis and cs-ELISA (Fig. 7A and Fig. S7A). GlialCAM variants, except GlialCAM-ΔC, supported the PM expression of MLC1-wt (Fig. 7A) indicating that the C-tail of GliaCAM is sufficient to rescue MLC1 folding/trafficking *in trans*. Expression of the GlialCAM C-tail in the CD4t-GCAM_Ct_ chimera was able to restore the PM expression of the MLC1-wt, in contrast to the full-length CD4 or the truncated CD4, lacking its C-terminal tail (CD4t)^28,43^ (Fig. 7B). CD4t-GCAM_Ct_ overexpression also delayed the ER clearance of MLC1-wt in the presence of BFA (Fig. 7C). Confirming these results, recombinant GST-GlialCAM_Ct_ was able to capture MLC1-wt, P92S and C326R in a pull-down assay (Fig. 7D).

To examine the chaperone-like consequences of GlialCAM structural variations outside the ER, we determined the morphology of the endo-lysosomal compartment as in Fig. 4A and S4A,B. Importantly, expression of GlialCAM-ΔC in GlialCAM-depleted cells induced enlarged accumulative Lamp + lysosomal compartment and was not unable to rescue the endosomal compartment homeostasis/proteostasis stress (Fig. 7E, Fig. S7B). These data revealed that the C-terminal cytosolic tail of GlialCAM can promote conformational maturation and assembly of the signalling complex by rendering MLC1 resistant to ERAD.

Finally, to directly address the GlialCAM impact on the stability of the newly synthesized MLC1 molecules, we performed metabolic pulse labelling with [^35^S]-methionine and [^35^S]-cysteine. Overexpression of GlialCAM increased the biogenesis of MLC1-wt by > 2-fold during a short 20 min pulse labelling (Fig. 7F). This could attribute to both fast co- and posttranslational folding and delayed ER degradation of the MLC1-wt, considering that one MLC1 chain (377 amino acids) translation is completed in one min (~ 6 amino acids/s)^44^.

To more accurately estimate the conformational maturation efficiency of MLC1 and minimize the influence of ER degradation of GlialCAM on MLC1, we improve the signal intensity by implementing a longer pulse labelling (1 h), followed by a 3 h chase. This approach encompasses the labelling of a larger cohort of newly synthesized proteins, as well as monitoring the combined effect of three consecutive ER processes: MLC1 degradation, folding, and ER-exit. It also serves as an indicator to membrane protein processing along the biosynthetic pathway because MLC1 lacks N-glycosylation typically used to detect the processing-dependent mobility shifts of secreted polypeptides^45,46^. The increased radioactive ^35^Met and ^35^Cys incorporation into the wt and mutant MLC1 after 3 h chase indicates that GlialCAM overexpression enhanced the accumulation of the MLC1 variants in post-ER compartments (Fig. 7F,G, Fig. S7C) providing direct evidence of improved conformational maturation and diminished ER degradation of MLC1 molecules.

## Discussion

**Figure 8:**
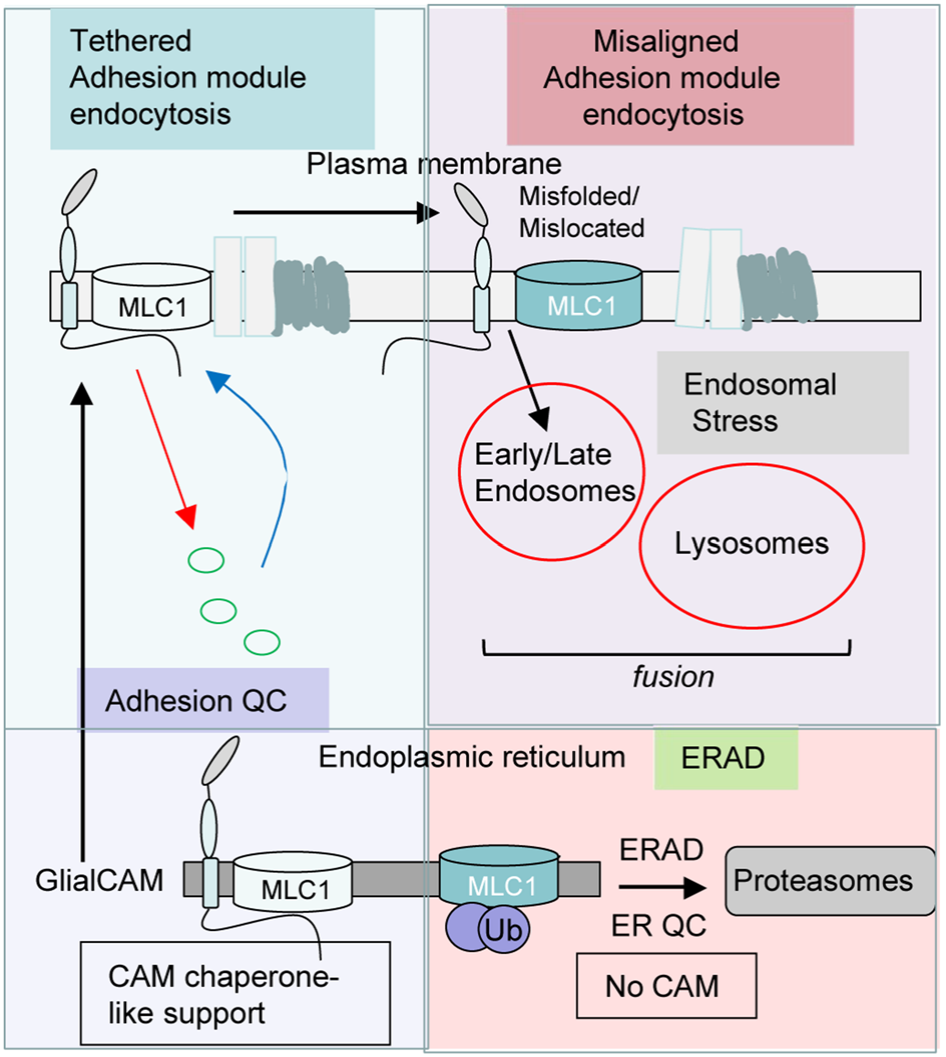
Cell adhesion molecule (CAM) can act as a chaperone-like molecule to regulate proteostasis and endocytosis. GlialCAM functions as a chaperone-like molecule in the ER by ensuring the conformational maturation of the regulatory membrane protein MLC1 and consequently protects MLC1 against ERAD. As a result, GlialCAM enhances the ER-exit, the PM targeting and the tethering of MLC1 variants. At the PM, GlialCAM regulates the diffusional phase separation of the PM proximity adhesion module, which links GlialCAM to early endocytosis dynamics of at least cluster members MLC1 and chloride channel ClC-2. Misaligned adhesion cluster, probably partly due to the inappropriate stoichiometry upon MLC1 or GlialCAM misfolding/mislocation or lack of expression leads to loss of proteostasis health at the late endosomal/lysosomal compartment. Hence, CAM can shape the dynamics of membrane protein folding QC, the diffusional phase separation at the PM and endocytosis of the proximity adhesion cluster.

Here we found that GlialCAM acts as a PQC factor both at the ER, as well as along the endocytic compartments (Fig. 8). In the ER, GlialCAM exhibits an obligatory chaperone-like activity toward the newly synthesized MLC1 nascent chains by aiding their conformational maturation/stabilization and protecting both the wt and mutant MLC1s against polyubiquitination- and proteasomal-dependent ERAD. Our biochemical turnover studies rule out the possibility that GlialCAM/MLC1 heteromerization acts on the MLC1 biogenesis by masking the ER-retention motif and/or exposing the ER-export signal in MLC1. Jointly, these results provide a mechanistic framework for the absolute dependency of the MLC1 heteromeric signalling cluster on GlialCAM expression. The indispensable role of GlialCAM in the biogenesis of the MLC1 signalling cluster with its endosomal homeostatic function offers a plausible explanation for the variety of brain dysfunctions associated with this cluster^15–17,47–50^ and highlights the broader role of CAMs in maintaining cellular health.

Distinct molecular events have been identified for the mechanism and function of CAMs heterooligomerization. The ER exit of the multimeric neuronal synaptic N-Methyl-D-aspartic/aspartate receptor (NMDA) requires two NCAM-subunits to mask its ER retention signal and enhance the PM delivery of the NMDA^51,52^. NCAM is also known to modulate the synaptic trafficking of the NMDA receptor^51,52^. The Ig-domain type CAMs neuroplastin and basigin interact with the PM Ca^2+^-ATPases (PMCA) 1–4 subunits and facilitate the PMCA-complex delivery to the cell surface independently of their Ig domains. At the PM, the interaction modulates both the Ca^2+^ transport activity and Ca^2+^ binding-site exposure to the cytoplasm^53^. Based on earlier data, we can rule out the transcriptional regulation of MLC1 by GlialCAM^12^.

Our findings provide direct evidence in support of the chaperone-like folding assistance of CAM for MLC1 variants and be a QC-factor for the MLC1 signalling complex. The cytosolic C-terminal tail of GlialCAM promotes the conformational maturation/assembly of the MLC1 in the ER. Whether the association of GlialCAM with MLC1 takes place after their nascent chains synthesis or initiated cotranslationally as demonstrated for certain oligomeric proteins^54^, remains to be tested.

The data that GlialCAM disease R92W mutation in the N-terminal IgV adhesion domain has reduced PM stability agree with a requirement of extracellular adhesion of GlialCAM to position MLC1 at the PM^27,55^. GlialCAM partitions the PM signalling cluster and the misaligned transmembrane phase separation seems to contribute to interfered interactions of signalling complexes in GlialCAM expressing cells both in trans and cis^55^. Notably, the neuroplastin CAMs have a similar anchoring role for the GluR1, GABA_A_, and monocarboxylate transporters^56,57^. Our model suggests that GlialCAM regulates the endocytosis dynamics of the MLC1 cluster members consistent with its influence on the transmembrane signalling^1^, the formation of tight junctions, and cell polarity^33^. Here, it modulated the PM-endosomal resident time of MLC1 and ClC-2, explaining the GlialCAM-mediated regulation of the ClC-2 current density^12^.

Intriguingly, the primary expression defect of MLC1 mutants (e.g. P92S or S280L) or as a consequence of GlialCAM loss-of-expression, here we report an accelerated drop in the vesicular pH along with the endo-lysosomal compartments (Fig. 4D,E), suggesting that the vacuolar (H^+^) V-ATPase responsible for acidification is incorporated and functional. The endosomal compartment enlargement is likely due to an increased propensity of homo- and heterotypic fusion of endosomes as suggested by the redistribution of the biochemical hallmarks of endo-lysosomal organelles (Fig. 4A–C). This could be, at least partly, due to the elevated cytoplasmic Ca^2+^ concentration, which is accompanied by augmented lysosomal exocytosis and activation of a proteotoxicity-dependent PQC mechanism^32^. Consistent with the critical role of adhesion in proteostasis health, a recent study showed that cell–cell adhesion enhances cellular resistance against proteotoxicity^58^. The augmented fusion and/or reduced fission propensities of endo-lysosomes with accelerated acidification of endocytic compartments may have far-reaching consequences in a variety of signalling processes involved in brain homeostasis.

By extrapolation, our results also suggest possible alterations in several ubiquitinated receptors and PM protein trafficking, as well as the downstream cellular signalling connected to the MLC1 signalling cluster. Substantial changes were observed in the sorting kinetics of ubiquitinated and recycled transmembrane cargoes and the fluid-phase markers in cells expressing mutant MLC1 (Fig. 4). While the precise molecular explanation requires further research, the observed phenomena could be, at least partly, explained by perturbations of the vesicular pH regulation via modulated counterion conductances and/or subunit compositional changes of the V-ATPase, as well as the rearrangement of the actin cytoskeleton as observed in MLC1 haploinsufficient cells^15,16^. Based on these considerations we propose that the MLC1-heteromer formation plays a critical role in maintaining the endo-lysosomal compartment morphological and functional homeostasis and identities.

## Materials and methods

### Cell culture and transfection

Parental or inducible Lenti-X Tet-On^5^ HeLa and U251N cells with and without 2HA-MLC1 expression were used in experiments and cultured under standard conditions. Lentivirus production, transduction, doxycycline induction^5^ and depletion of endogenous GlialCAM expression were done as described before^11^. Transfection of plasmid DNA was performed using Lipofectamine 2000 (Thermo Fisher Scientific) and siRNA using RNAiMAX or Oligofectamine transfection reagent (Thermo Fisher Scientific). Primary rat astrocytes were isolated and cultures and transfected as described before^11^.

### Live-cell surface ELISA

The membrane protein expression, turnover and recycling were measured using the PM epitope labelling and cs-ELISA in live cells for kinetic studies^5,12,28,59^. Internalization was measured for 5 min, recycling 20 min and stability for indicated times at 37 °C. Transferrin-HRP or HRP-conjugated secondary antibody (Ab) was measured either by luminescence using HRP-Substrate (SuperSignal West Pico, Thermo Fisher Scientific) or Ampilite (ATT Bioquest, CA, USA) or Amplex Red assay (Thermo Fisher Scientific).

### HRP-uptake and recycling assay

The enzyme horseradish peroxidase (HRP; Thermo Fisher Scientific) was loaded in 0.1 mg/ml for indicated times into cells at 37 °C. Cells were lysate and HRP signal was detected using Ampilite (ATT Bioquest, CA, USA). The received signal was normalized to mg of protein in each sample. For measuring recycling capacity, HRP was loaded for 10 min and allowed to recycle for 20 min at 37 °C before measurement from the medium.

### Immunoprecipitation and protein analyses

In total cell IP, the Ab was added directly to cell lysates. Selective isolation of GlialCAM from the PM was achieved by cs-IP using anti-GlialCAM Ab (1:2000, R&D Systems, MN, USA). Ab was bound on ice to the live cell PM for 45 min, after which the unbound Ab was washed off. Cells were lysed in Triton X-100 lysis buffer (1% Triton X-100, 25 mM Tris–Cl, 150 mM NaCl, pH 8.0, 10 μM MG132 containing 20 μM PR-619, 10 μg/ml pepstatin + leupeptin, 1 mM phenylmethylsulfonyl fluoride, and 5 mM *N*-ethylmaleimide) on ice or in co-IP assays, a milder lysis buffer was used by changing detergent to 0.4% NP-40. Reagents were from Sigma. Precipitates were isolated using magnetic beads (Dynabeads M-280, ThermoFisher Scientific). For detecting direct ubiquitination of MLC1 or GlialCAM, lysates were denatured using 1% SDS for 5 min, after which the SDS concertation was adjusted to 0.1%. The second IP step was performed using anti-HA (Biolegends, CA, USA; for MLC1) or anti-GlialCAM (Santa Cruz, TX, USA and ThermoFisher Scientific). P4D1 (Santa Cruz, TX, USA) anti-ubiquitin Ab was used to detect ubiquitinated conjugates. Treatment with cycloheximide (100 μg/ml, Sigma) was carried out in full medium at 37 °C for indicated times. BFA treatment (5 μg/ml, Sigma) was done in the full medium at 37 °C for 20 h. Proteasomes were inhibited using Bortezomib (1 μM, 4 h, LC Laboratories, MA, USA), lysosomes with leupeptin-pepstatin (1 μg/ml, o/n, Sigma) and autophagy with 3-methyladenine (5 μM, 4 h, Sigma). For all assays, polypeptides were separated using SDS-PAGE and Western blot analysis. Densitometric analyses were done using Image Studio Lite (LiCOR) or Fiji (National Institutes of Health). When quantification was done for the IP samples, the signal was normalized to the amount of precipitated MLC1 or GliaCAM.

### Metabolic pulse-chase analyses

Metabolic pulse-chase experiments were performed essentially as described^46^. Briefly, control and GlialAM overexpressing cells were pulse-labelled with 0.2 mCi/ml ^5^S-methionine and ^35^S-cysteine (EasyTag Express Protein Labeling Mix, PerkinElmer) in cysteine and methionine-free medium for 20 min or 1 h at 26 °C (incorporation efficiency), and with 3 h chase at 37 °C (maturation efficiency) when indicated. MLC1 was immunoprecipitated with an anti-HA antibody. Incorporated radioactivity was visualized by fluorography and densitometric phosphorimager analysis using a Typhoon imaging platform (GE Healthcare).

### Microscope imaging

For confocal colocalization microscopy (Leica TCS SP8X confocal microscope or LSM780 microscope, Carl Zeiss MicroImaging, 63 ×/1.4 NA Plan Apochromat oil-immersion objective) cells were cultured in 100 µg/ml poly-l-lysine-coated coverslips and fixed with 4% paraformaldehyde in PBS for 15 min. Intracellular antigens were visualized in fixed, permeabilized cells using the indicated primary Abs in PBS-0.5% BSA for 1 h at room temperature. Primary antibodies were mouse anti-HA (Biolegends, CA, USA), rabbit anti-EEA1 (Cell Signalling, MA, USA), rabbit anti-calreticulin, mouse anti-LAMP2 (Developmentals studies hybridoma bank, IA, USA) and anti-LAMP1 (Abcam, Cambridge, UK). Secondary antibodies goat anti-rabbit Alexa 488 and 594 were from ThermoFisher Scientific. Image analysis was performed using Fiji software.

To measure the lateral diffusional mobility of MLC1 at the PM, the FLIP experiment was performed using a confocal microscope. All imaging and bleaching were carried out using the 488-nm laser line. Following the acquisition of a baseline image, a region was selected for photobleaching. Time-series images were acquired from the adjacent area to photobleached one for monitoring loss in intensity. Typically, 3–6 intensity curves per experiment were collected and the final curve represented in figures are the averages of normalized intensities from at least three different experiments. The decay rates were analyzed using GraphPad Prism.

The high-content screening was performed using a widefield In Cell 2200 Imaging Analyzer equipped with an sCMOS camera and LED light source (GE Healthcare Life Sciences, USA; 60 ×/0.95 Plan Apo objective). Early endosomes were labelled with anti-EEA1 and late-endosomes, lysosomes anti-Lamp2 as above and nucleus using DRAQ5 (ThermoFisher Scientific). The nuclear stain was used to count an equal amount of cells (~ 300) from three experiments and image analysis was performed in In Cell Developer Toolbox (GE Healthcare Life Sciences, USA) by using a colocalization module to identify EAA1/Lamp2 positive endosomes with overlap coefficient and segmentation module to measure Lamp2 lysosome size and number. Statistical analysis was done in GraphPad Prism.

The pH of endo-lysosomal organelles was measured using live-cell single vesicle fluorescence ratio image analysis as described previously^5,28,37,38^. The FITC-dextran (10 kDa, ThermoFisher Scientific) was loaded to live cells for 5 min at 37 °C, washed and the remaining label was chased for indicated times to measure the intraluminal pH at the length of the endo-lysosomal pathway. Model membrane proteins (CD4tl-L57C and CD4tl-Ub)^28^ were labelled sequentially with mouse anti-human CD4 (Biorad) and with FITC-goat anti-mouse secondary Fab (Jackson Immunoresearch) and allowed to continue for indicated times at 37 °C. Recycling endosomes were labelled with 5 µg/ml FITC-Tf (Jackson Immunoresearch) for 1 h. At least > 250 vesicles from 25–50 cells per experiment were analyzed, and the average weighted mean was calculated from the three or more independent experiments. The analysis was performed on an inverted fluorescence microscope Nikon TI-E equipped with Lumencor Spectra X light source and electron-multiplying charge-coupled device (Photometrics) equipped with an Evolve 512 electron-multiplying charge-coupled device (EM CCD) camera (Photometrics Technology) and a 63 ×/1.4 numerical aperture (NA) Plan Apochromat oil-immersion objective. The acquisition was performed at 490 ± 5- and 440 ± 10-nm excitation wavelengths using a 535 ± 25-nm emission filter and was analyzed with NIS-Elements (Nikon).

### Recombinant protein production and GST pulldown assay

GST-GCAM expression in *E. coli* BL21 was induced with 0.1 mM IPTG for 5 h at 30 °C, and bacteria was lysed in (100 mM Tris–HCl pH 7.5, 50 mM NaCl, 5 mM EDTA, 0.5% Triton X-100, 5% glycerol, 4 mM DTT, protease inhibitors and 1 mM PMSF) and affinity purified on glutathione-Sepharose beads (GE Healthcare). Hela cell lysates (0.4 mg) expressing MLC1 molecules were used to detect interaction with the GlialCAM tail and GST alone was used as control. Glutathione-Sepharose bound GST-GCAM was incubated with lysates for 2 h at 4 °C, beads were washed, and bound forms were analyzed using SDS-PAGE and Western blot analysis.

### Statistical analysis

All data analysis was performed using GraphPad Prism 8.0–9.0 (San Diego, CA, USA). Paired or unpaired two-tailed Student’s *t* test was used for p-values as indicated in the figure legends. All data in curves and bar plots represent the means average of at least three or more independent experiments. Repeat numbers and parametric *t *test p-values for individual assays with comparison to Mann–Whitney test, when possible, can be found as Supplementary Table S1 online. Data are expressed as means ± SEM. ****p < 0.0001, ***p < 0.001, **p < 0.01 and *p < 0.05.

## Supplementary Information

Supplementary Figures.

Supplementary Table S1.
