# Supplementary material for: Control of membrane protein homeostasis by a chaperone-like glial cell adhesion molecule at multiple subcellular locations
Source: Sci Rep. 2021 Sep 16;11:18435. doi: 10.1038/s41598-021-97777-4 (PMC8446001; doi:10.1038/s41598-021-97777-4)
Supplement: Supplementary file 1 — Supplementary Figures. [file 41598_2021_97777_MOESM1_ESM.pdf]

Supplemental information

Control of membrane protein homeostasis by chaperone-like glial cell adhesion molecule at multiple subcellular locations

Haijin Xu[5], Sandra Isenmann[3,4], Tania López-Hernández[7,8], Raúl Estévez[7,8], Gergely L. Lukacs*[5,6], Pirjo M. Apaja*[1,2,3,4]

[1]University of Adelaide, Department of Molecular and Biomedical Sciences, Adelaide 5005, South Australia, Australia
[2]Flinders University, College of Public Health and Medicine, Molecular Biosciences Theme, Bedford Park 5042, South Australia, Australia
[3]Organelle Biology and Disease, South Australian Health and Medical Research Institute, Adelaide 5000, South Australia, Australia
[4]EMBL Australia, Adelaide 5000, South Australia, Australia
[5]Department of Physiology and Cell Information Systems, [6]Department of Biochemistry, McGill University, Montréal, Quebec H3G 1Y6, Canada.
[7]Unitat de Fisiologia, Departament de Ciències Fisiològiques, IDIBELL-Institute of Neurosciences, Universitat de Barcelona, L'Hospitalet de Llobregat, Spain
[8]Centro de Investigación en Red de Enfermedades Raras (CIBERER), ISCIII, Madrid, Spain.

*** Correspondence:**
P. M. Apaja
University of Adelaide, SAHMRI
5005 North Terrace, Adelaide, South Australia, Australia
p.apaja@adelaide.edu.au
Ph: +61-812-84931
*G. L. Lukacs
Department of Physiology, McGill University
3655 Promenade Sir-William-Osler, Montreal, Quebec H3G 1Y6, Canada
gergely.lukacs@mcgill.ca
Ph: +1-514-398-5582

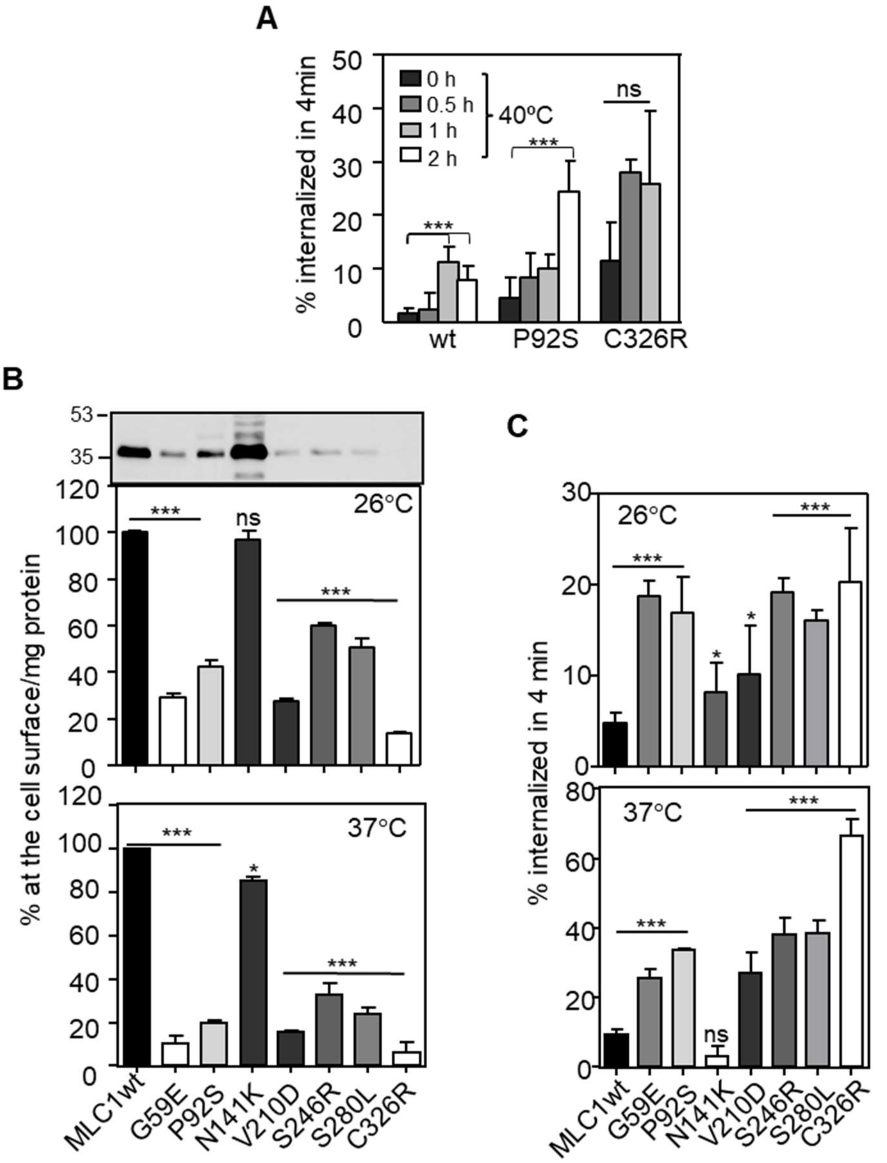

Figure S1. **Disease associated MLC1 mutants are misfolded and unstable at the PM.** (A) Effect of temperature-induced unfolding on MLC1-wt and misfolded MLC1 (P92S, C326R). Cells were temperature rescued at 26ºC to enhance folding of MLC1 and then unfolded at 40ºC for indicated times, after which the internalization was measured at 37ºC for 4min using cs-ELISA. (B) Expression of different MLC1 diseases associated mutations at the PM (B) and the effect of misfolding on internalization (C) were measured using cs-ELISA. Cells were temperature rescued at 26ºC to enhance folding or unfolding at 37 ºC. A summary is in Fig.1D. Means ± SEM, n≥3, p-value: *<0.05, *** <0.001 are calculated to MLC1-wt in each panel.

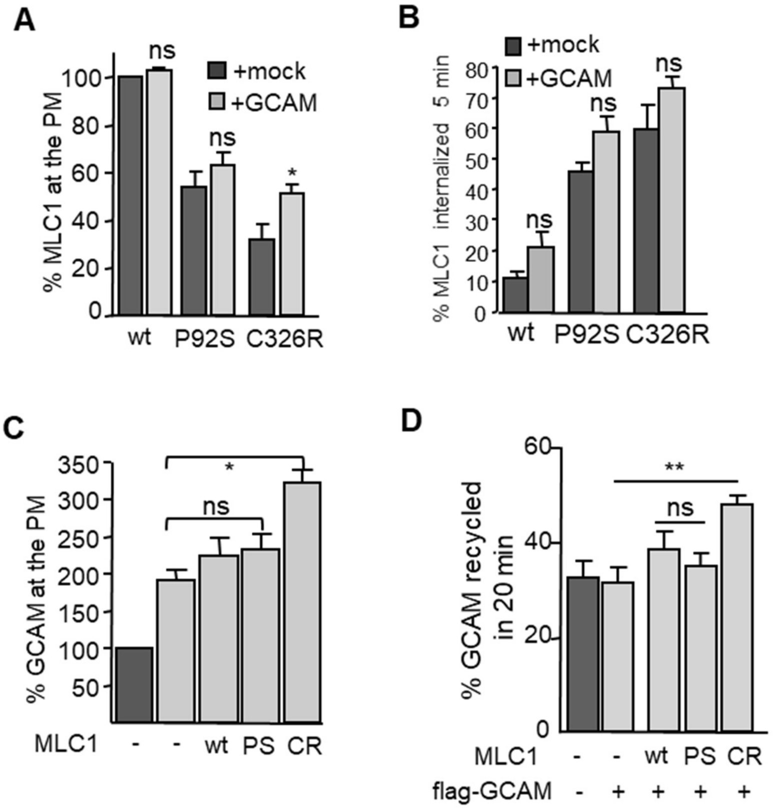

Figure S2. **Crosstalk effect of MLC1 mutants on GlialCAM endosomal trafficking.** (A) Cs-ELISA of MLC1 amount at the PM in Hela cells with or without GlialCAM overexpression. (B) GlialCAM effect on MLC1-wt and misfolded mutant internalization. Internalization (5min) was measured using cs-ELISA in Hela cells. GliaCAM slightly increased MLC1 internalization. (C) The effect of transiently overexpressed MLC1 variants on the PM expression of the endogenous GlialCAM. Cell surface expression of GlialCAM was measured by cs-ELISA and expressed as relative to mock-transfected Hela cells. n=3 *$p<0.05$. (D) MLC1 overexpression effect was measured on GlialCAM recycling by labelling GlialCAM at the PM with anti-GlialCAM and then internalized for 10min at 37°C. Recycling was induced for 20min at 37°C after blocking the remaining PM expression with Fab and measured using cs-ELISA in Hela. n=3 **$p<0.01$, ns; non-significant.

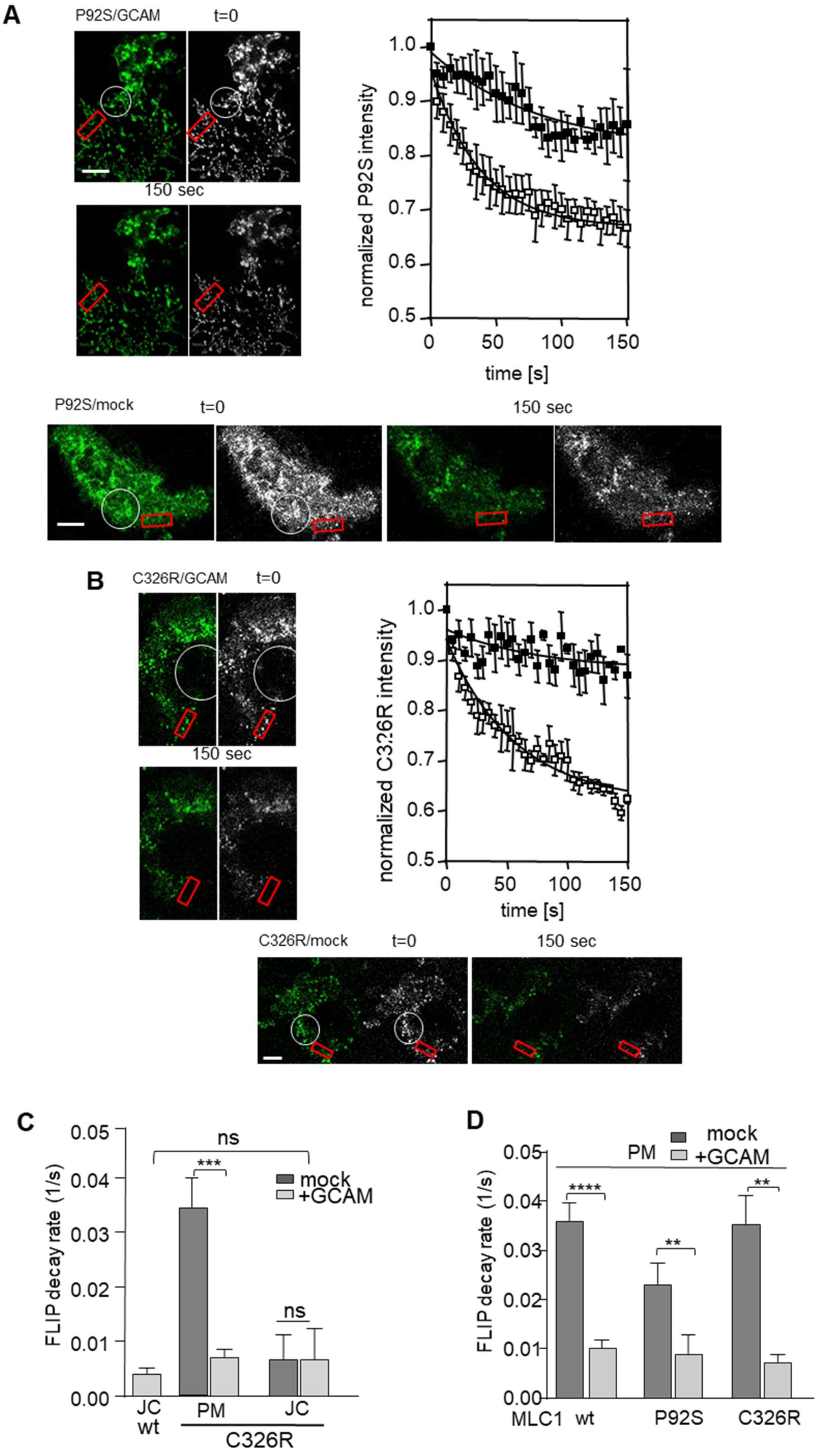
A
P92S/GCAM
t=0
150 sec
normalized P92S intensity
time [s]
P92S/mock
t=0
150 sec
B
C326R/GCAM
t=0
150 sec
normalized C326R intensity
time [s]
C326R/mock
t=0
150 sec
C
FLIP decay rate (1/s)
ns
***
mock
+GCAM
ns
JC
wt
PM
JC
C326R
D
PM
mock
+GCAM
FLIP decay rate (1/s)
****
**
**
MLC1 wt
P92S
C326R

Figure S3. **GlialCAM regulates the lateral phase diffusion of MLC1.** (A-B) FLIP to measure disease associated MLC1-P92S and C326R lateral diffusion at the PM. Indicated areas (white circles) were photobleached and the adjacent areas (red squares) loss in fluorescence were monitored. The normalized intensity traces of fluorescence decay in cells with or without GlialCAM overexpression are shown. The summary of P92S decays is in Fig.6D. (C) Summary of C326R decay rates. PM; free PM and JC; cell-cell junctional area. (D) Summary of MLC1-wt, P92S and C326R decay rates at the free PM areas (PM) in endogenous and GlialCAM overexpressing cells. Means ± SEM, n≥5, ns; non significant, ** < 0.01, *** <0.001, ****<0.0001.

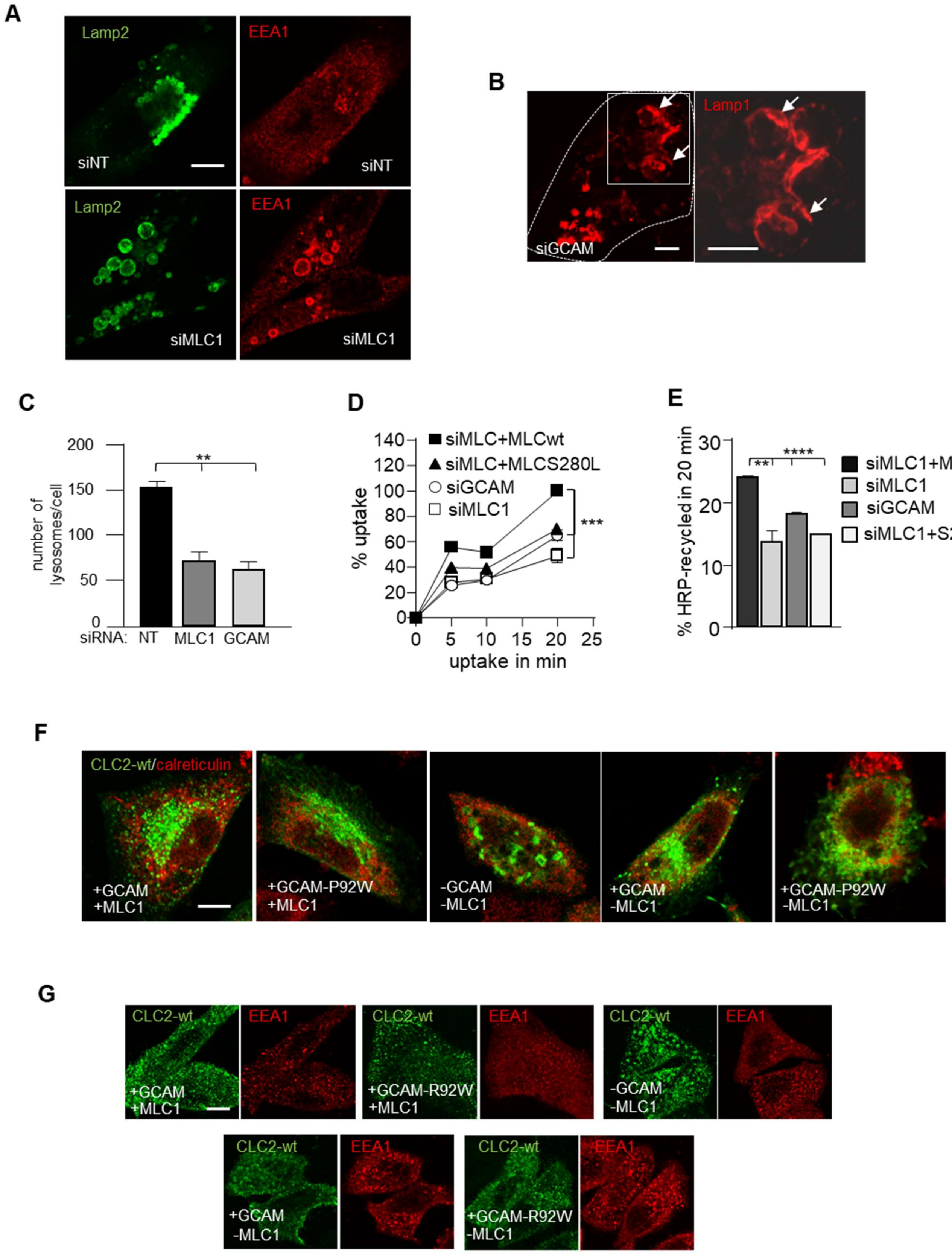

Figure S4. **A misaligned GlialCAM signalling cluster causes late endosomal enlargement**

(A) MLC1 expression effect on late endosomes-lysosomes was monitored using siMLC1 and

siNT (non-targeted) mediated depletion in astrocytic U251N cells. Lamp2 marker is for lysosomes and EEA1 for early endosomes. Colocalization is in Fig. 4A. Bar: 5 µm. (B) Immunofluorescence confocal microscopy of lysosomal morphology in GlialCAM depleted U251N cells. Arrows and insets show a magnification of enlarged $LAMP1^{+}$ lysosomes. Bar 2 µm. (C) High-content image analysis of average lysosome number per cell from 4B-C. siNT was used as a control in comparison to siMLC1 and siGCAM depleted U251N cells (~300/repeat, n=3). (D) HRP-uptake was measured in siNT, siMLC and siGCAM astrocytic U251N cells after indicated time points using fluorogenic substrate and a fluorescence microplate reader. Signal was normalized to mg of protein and 30min point of siNT was defined as 100%. (H) As in D, but recycled HRP was measured after 20min from the medium. (F) Immunofluorescence confocal microscopy of chloride channel ClC2 subcellular location in HeLa cells overexpressing GCAM-wt or mutant P92W or depleted of GlialCAM with (+) or without (-) MLC1-wt expression. Calreticulin was used as an ER marker. Bar 5 µm. (G) Immunofluorescence of internalized ClC2 with EEA1 in cells +/- GCAM and +/- MLC1 overexpression in HeLa cells. Colacalization is in Fig.4H. Bar: 5 µm. $n \geq 3$, $^{**}p<0.01$, $^{***}p<0.001$, $^{****}p<0.0001$, ns; non-significant.

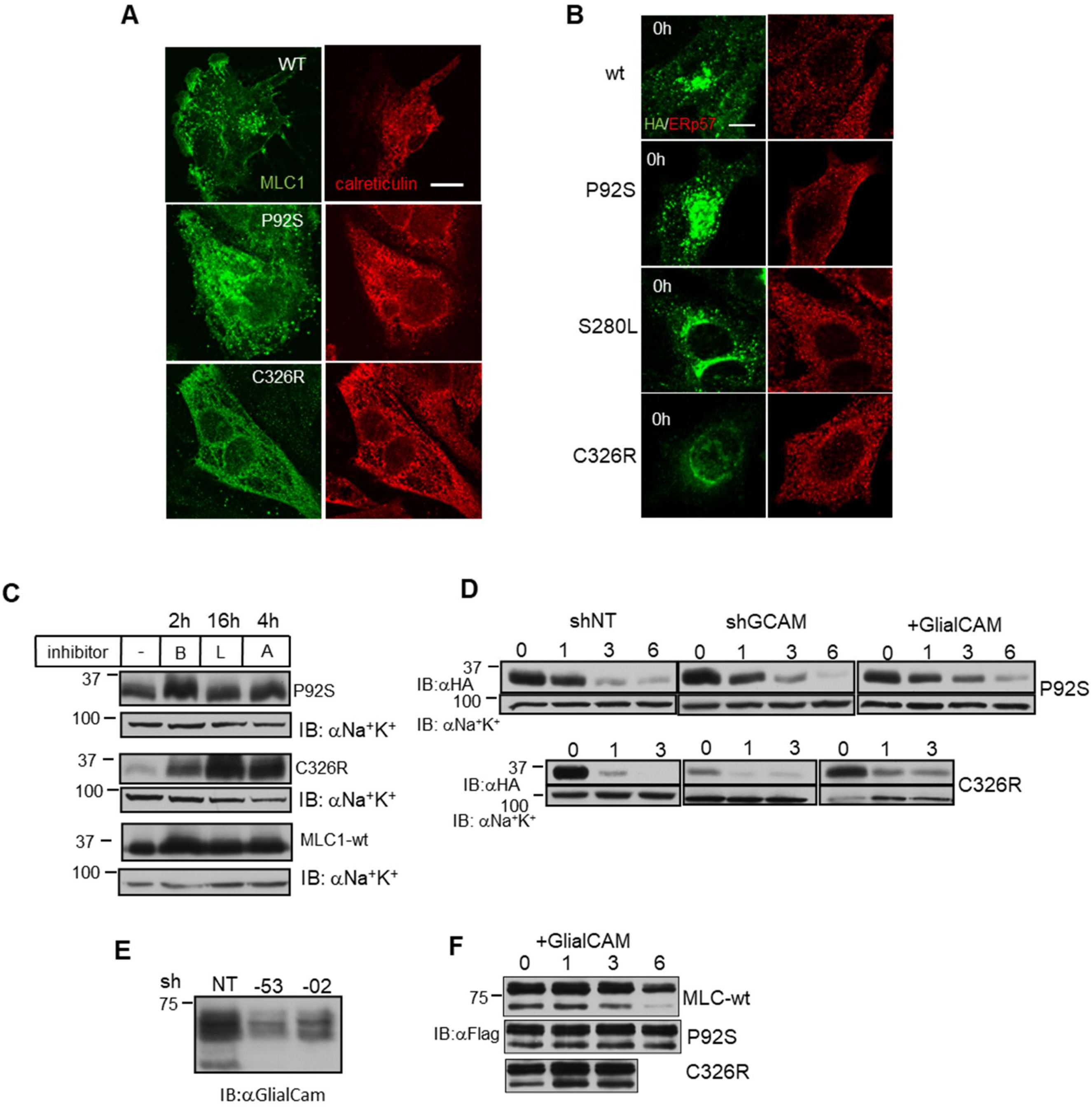

Figure S5. **GlialCAM effect on disease associated MLC1 mutants degradation kinetics.** (A) Immunofluorescence of MLC1 variants with the ER marker calreticulin. MLC1 variants were detected with indirect immunostaining using an anti-HA primary antibody in astrocytic U251N. Colocalization is in Fig.5A. Bar 5 μm. (B) Immunostaining of MLC1 variants before (0min) CHX chase. MLC1 was detected with an anti-HA antibody and ERp57 was used as an ER marker. Colocalization is in Fig.5C. Bar 5 μm. (C) Inhibition of MLC1-P92S and C326R degradation using proteasome [B] inhibitor Bortezomib (1 μM) for 2h, and lysosome [L]

inhibitor leupeptin-pepstatin (5 μg/ml, 16h) and autophagosome [A] inhibitor 3-methyladenine (5 μM) for 4h. (D) The fast ER clearance was detected for MLC1-P92S, S280L and C326R during CHX-chase in comparison to disease associated mutants (relates to Fig. 3C). (E) Western blot of GlialCAM expression in non-target (shNT) or partially depleted (~60%) by two shGlialCAMs with distinct target sequences. (F) Expression of GlialCAM (+GlialCAM) for Fig.5D (MLC1-wt) and for S5D (P92S and C326R).

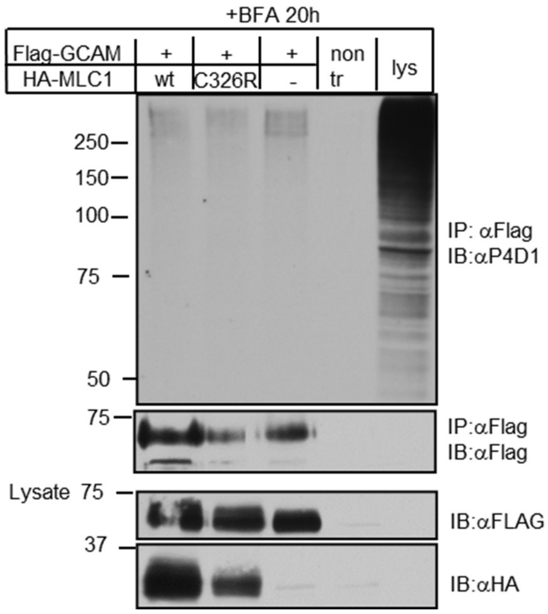

Figure S6. **MLC1 effect on GlialCAM ubiquitination in the ERAD pathway.** GlialCAM ubiquitination was measured in the ER in Hela cells treated with BFA for 20h and with or without MLC1-wt or C326R. GliaCAM was immunoprecipitated with anti-Flag in denaturing conditions to measure direct ubiquitination.

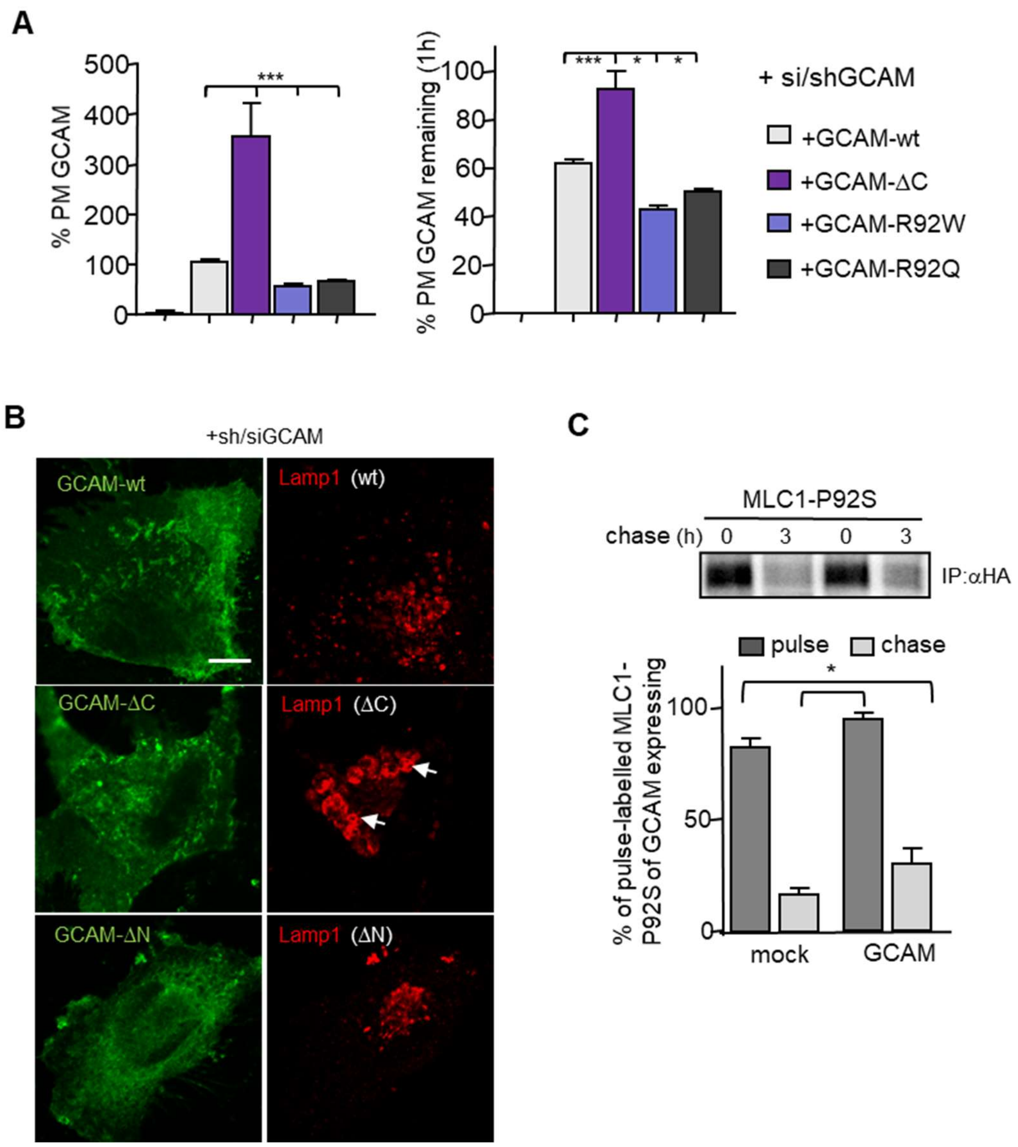

Figure S7. **GlialCAM regulates MLC1 early biosynthesis.** (A) The amount and the PM turnover of GlialCAM domains. Cs-ELISA of GlialCAM amount (left panel) and turnover (right panel) at the PM in cells depleted for GlialCAM and overexpressed for different GlialCAM regions. The deletion of the C-terminus stabilized the GliaCAM at the PM indicating the involvement of intracellular interactions. (B) The effect of GCAM truncation on the endo-lysosomal pathway morphology was monitored in GCAM depleted (si/sh) astrocytic U251N cells. GCAM expression was detected with anti-GCAM and lysosomes with anti-Lamp1 Ab. Arrow points to enlarged Lamp1+ lysosomes. Colocalization is in Fig.7E. Bar 5 µm. (C) Densitometer of metabolic 1h pulse at 26 °C and 3h chase at 37 °C of newly

synthesized MLC1-P92S with [^{35}S]methionine/cysteine in the presence or absence of GlialCAM overexpression. MLC1 was immunoprecipitated with an anti-HA antibody (relates to Fig.5). n=3, *p<0.05, ***p<0.001, ns; non-significant.

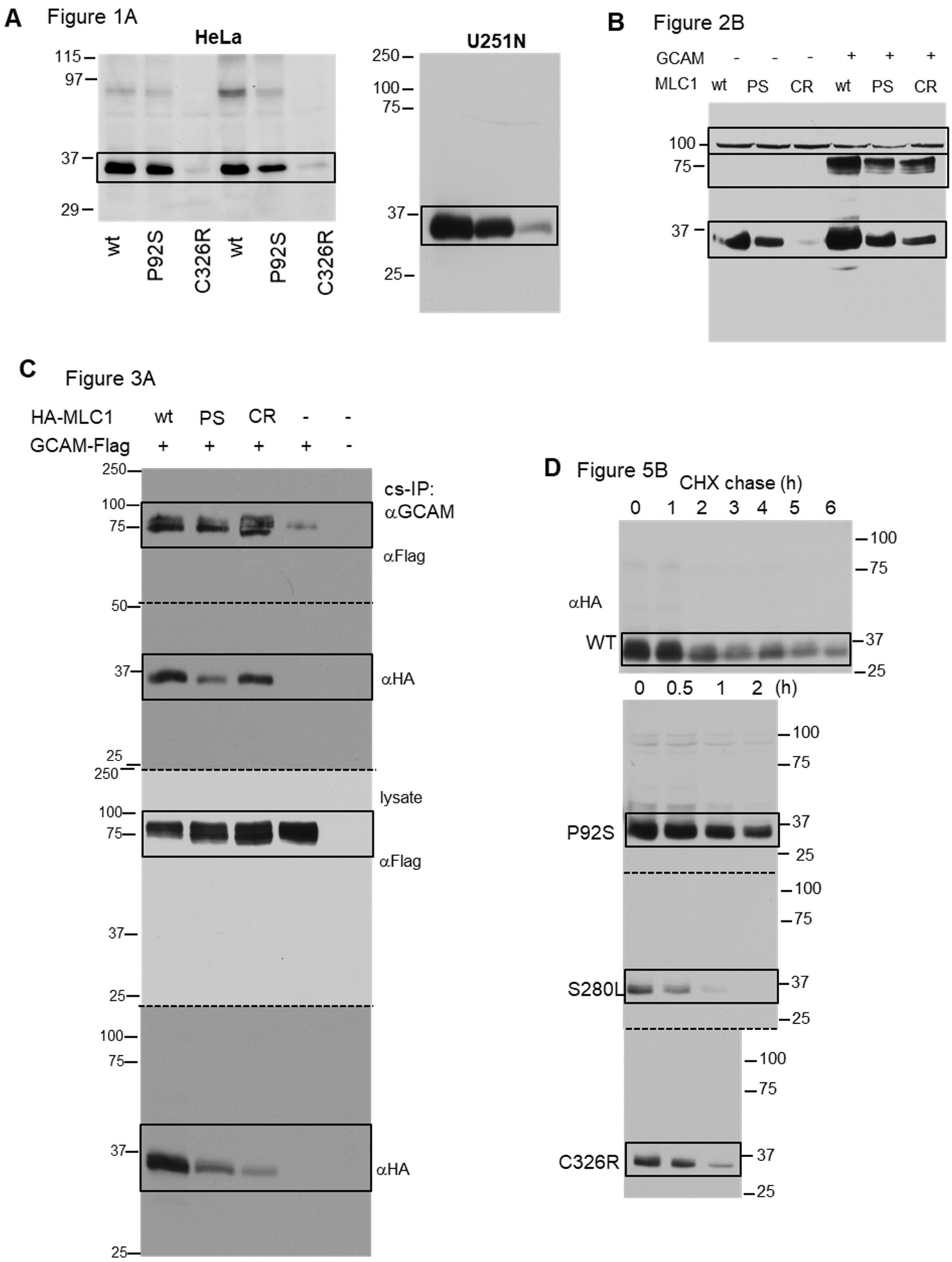

Figure S8. **Uncropped images of main figure sets.** Original immunoblots (A-M) and fluorograms (N-O). The black frame indicates the image used in the main figure, a dotted line separate gels or where the membrane was cut for probings.

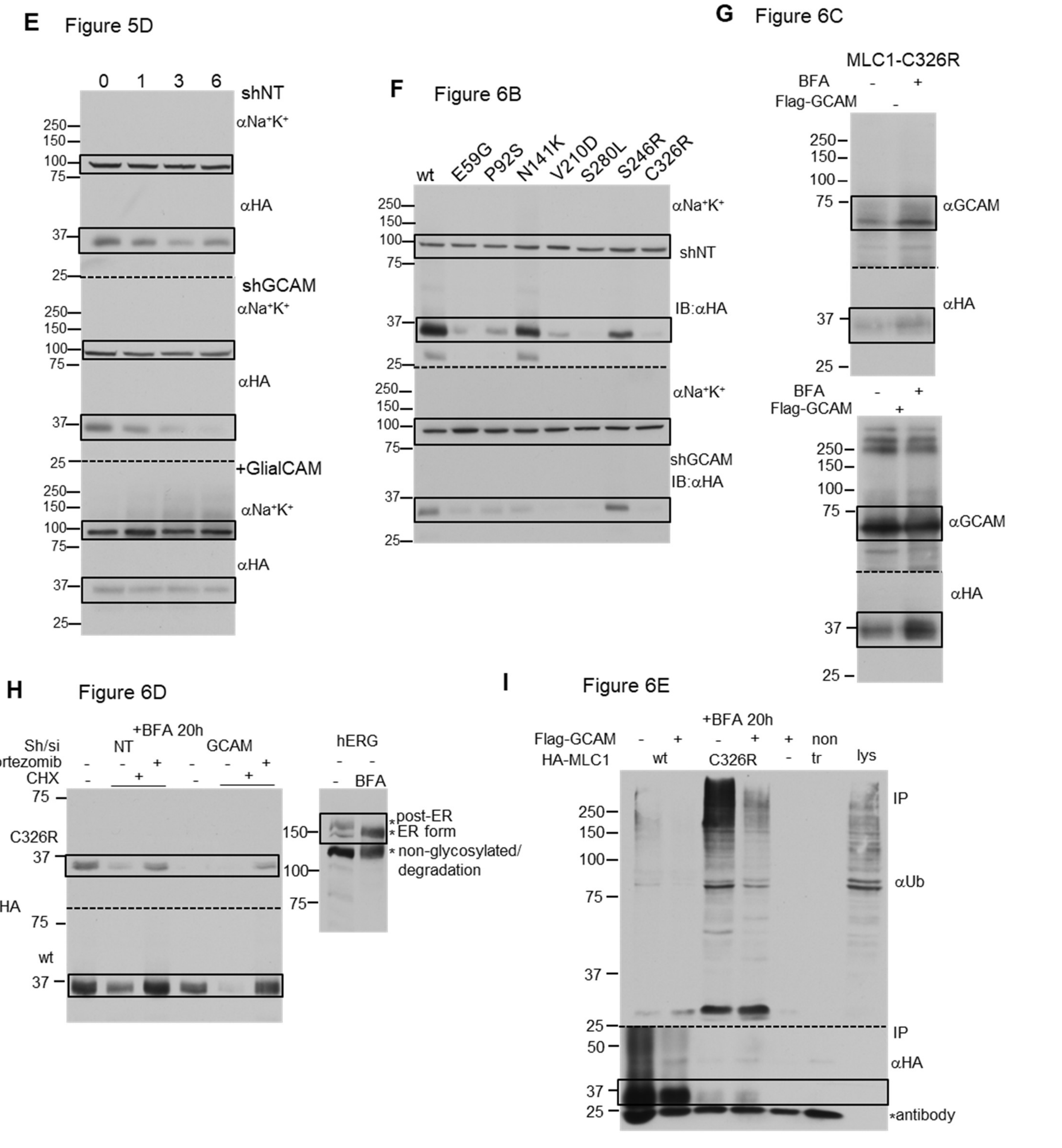

Figure S8. **Uncropped images of main figure sets (continue)**

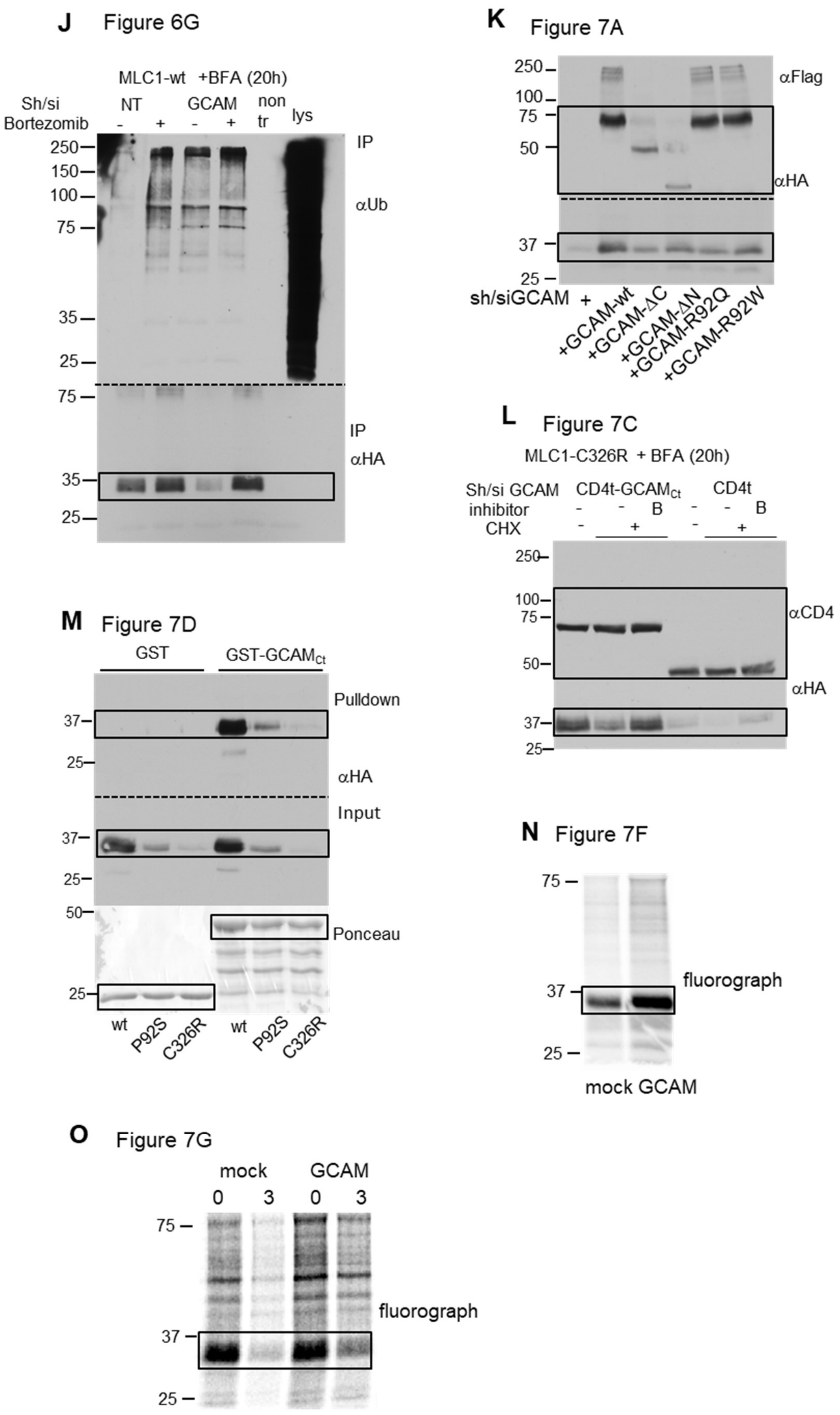

Figure S8. **Uncropped images of main figure sets (continue)**

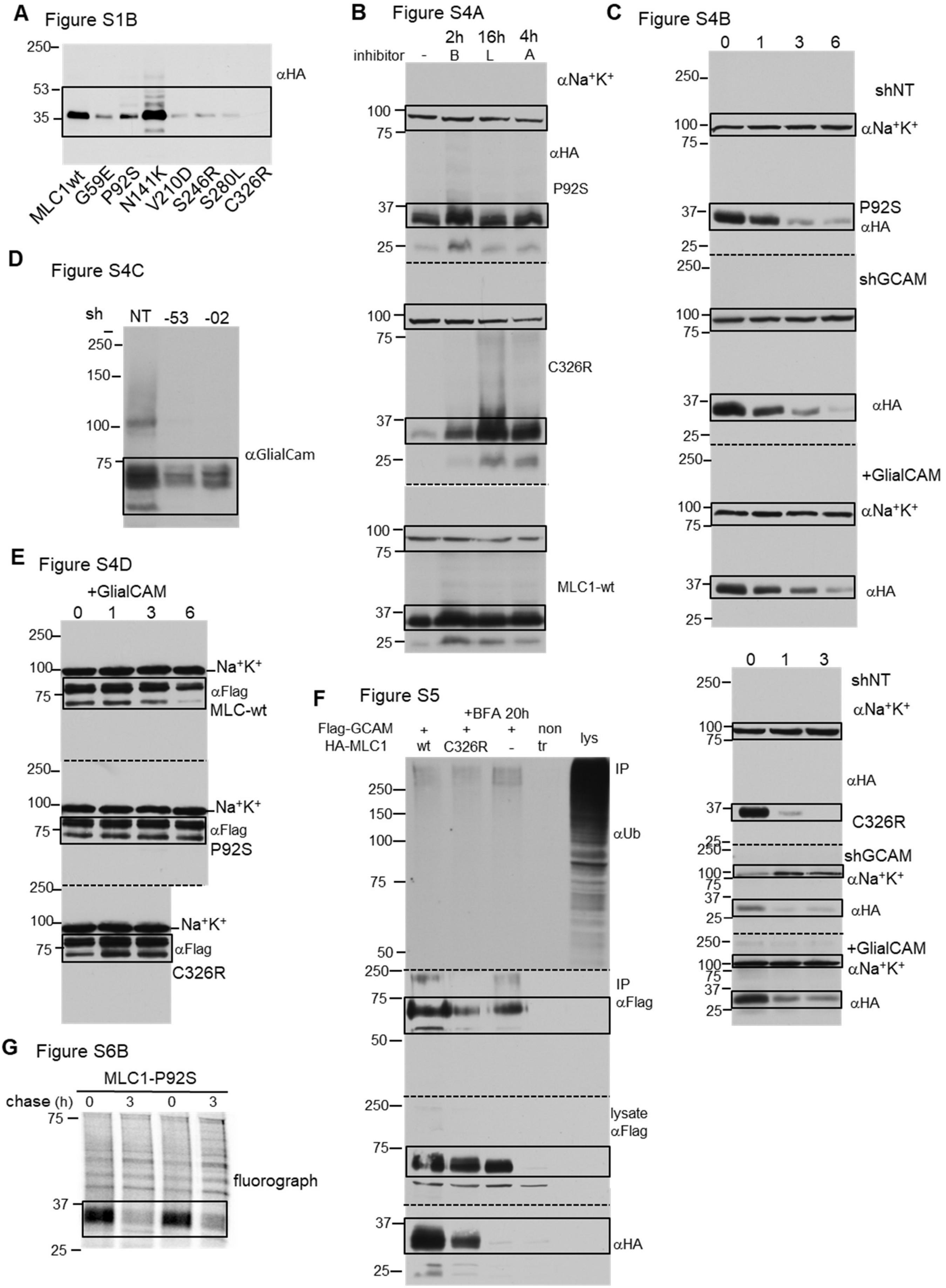

A Figure S1B
αHA
MLC1wt G59E P92S N141K V210D S246R S280L C326R
B Figure S4A
inhibitor - 2h B 16h L 4h A
αNa+K+
αHA
P92S
C326R
MLC1-wt
C Figure S4B
0 1 3 6
shNT
αNa+K+
P92S
αHA
shGCAM
+GlialCAM
C326R
D Figure S4C
sh NT -53 -02
αGlialCam
E Figure S4D
+GlialCAM
Na+K+
αFlag
MLC-wt
P92S
C326R
F Figure S5
+BFA 20h
Flag-GCAM
HA-MLC1
wt C326R - non tr lys
IP
αUb
αFlag
lysate
αHA
G Figure S6B
MLC1-P92S
chase (h) 0 3 0 3
fluorograph

Figure S9. Uncropped images of supplemental figure sets. Original immunoblots (A-F) and fluorogram (G). The black frame indicates the image used in the supplemental figure, a dotted line separate gels or where the membrane was cut for probings.
